# Sex-biased gene expression during neural differentiation of human embryonic stem cells

**DOI:** 10.3389/fcell.2024.1341373

**Published:** 2024-05-01

**Authors:** Philipp Pottmeier, Danai Nikolantonaki, Fredrik Lanner, Christiane Peuckert, Elena Jazin

**Affiliations:** ^1^ Department of Organismal Biology, Evolutionary Biology Centre, Uppsala University, Uppsala, Sweden; ^2^ Division of Obstetrics and Gynecology, Department of Clinical Science, Intervention and Technology, Karolinska Institute and Karolinska University Hospital, Stockholm, Sweden; ^3^ The Department of Molecular Biosciences, The Wenner-Gren Institute, Stockholm University, Stockholm, Sweden

**Keywords:** genetic sex differences, human embryonic stem cells, neural differentiation, KDM5D, UTY, X and Y chromosome, gametologs, transcriptomics

## Abstract

Sex differences in the developing human brain are primarily attributed to hormonal influence. Recently however, genetic differences and their impact on the developing nervous system have attracted increased attention. To understand genetically driven sexual dimorphisms in neurodevelopment, we investigated genome-wide gene expression in an *in vitro* differentiation model of male and female human embryonic stem cell lines (hESC), independent of the effects of human sex hormones. Four male and four female-derived hESC lines were differentiated into a population of mixed neurons over 37 days. Differential gene expression and gene set enrichment analyses were conducted on bulk RNA sequencing data. While similar differentiation tendencies in all cell lines demonstrated the robustness and reproducibility of our differentiation protocol, we found sex-biased gene expression already in undifferentiated ESCs at day 0, but most profoundly after 37 days of differentiation. Male and female cell lines exhibited sex-biased expression of genes involved in neurodevelopment, suggesting that sex influences the differentiation trajectory. Interestingly, the highest contribution to sex differences was found to arise from the male transcriptome, involving both Y chromosome and autosomal genes. We propose 13 sex-biased candidate genes (10 upregulated in male cell lines and 3 in female lines) that are likely to affect neuronal development. Additionally, we confirmed gene dosage compensation of X/Y homologs escaping X chromosome inactivation through their Y homologs and identified a significant overexpression of the Y-linked demethylase *UTY* and *KDM5D* in male hESC during neuron development, confirming previous results in neural stem cells. Our results suggest that genetic sex differences affect neuronal differentiation trajectories, which could ultimately contribute to sex biases during human brain development.

## Introduction

The importance of studying sex differences is today well recognized in many research fields, including neuroscience (Jazin and Cahill, 2010; Zagni et al., 2016; Golden and Voskuhl, 2017; Choleris et al., 2018). However, no studies have yet specifically addressed sex differences in multiple human embryonic stem cell lines (hESC). Models of stem cell differentiation are invaluable for this kind of research because they allow the study of genetic sex differences in the absence of sex hormones and their effects. Sex differences can have critical implications for the tissues they affect. In the developing nervous system, they have the potential to influence, e.g., the number of neurons, glial cells, synapses, and their organization. As a consequence, neural networks may develop that respond to stimuli in sex-specific ways (Arnold and McCarthy, 2016). Sex differences appear to be particularly relevant for several neurological diseases. Autism spectrum disorder, Tourette syndrome, and attention-deficit/hyperactivity disorder, for example, exhibit sex biases in symptoms, onset, and prevalence (Boyle et al., 2011; Loke et al., 2015; Tesic et al., 2019). These disorders are believed to have a neurodevelopmental origin, and it is likely that sex differences in early brain development act as a major contributor to the sex-biased development and manifestation of these diseases. Currently, sex differences in the human brain are largely attributed to the effects of sex hormones during critical periods of fetal development and adolescence (McCarthy, 2009; Arnold and McCarthy, 2016). However, an increasing body of evidence demonstrates that not only hormones alone play a role but also genetic factors, such as sex chromosome dosage compensation (Raznahan et al., 2018; Sidorenko et al., 2019; Warling et al., 2021), and differences in gene expression contribute to sex differences in the developing human brain (Carruth et al., 2002; Corre et al., 2016; Arnold, 2017; Gegenhuber and Tollkuhn, 2020; Cabrera Zapata et al., 2022). Despite the growing attention that research in sex differences receives, only a few studies specifically aim to investigate sex differences in embryonic stem cells, and to our knowledge, none of them include more than 1 cell line of each sex. Particularly, the role of sex-biased gene expression in early neuron development remains unclear. To address this gap, we collected four male and four female human embryonic stem cell lines, differentiated them into a mixed neuronal population over 37 days, and analyzed their gene expression profiles for sex differences using both total RNA sequencing and qPCR analysis. Our results reveal the presence of gene expression sex differences in all investigated stages of neuronal differentiation, but most pronounced after 37 days. We identified a number of candidate genes with potential contributions to sex-biased neuronal development. Finally, we demonstrate how overexpression of X-linked gametologous genes that escape X chromosome inactivation (XCI) is compensated by the expression of corresponding Y-linked homologs.

In summary, our research addresses a critical gap in understanding how sex differences in gene expression may influence early neuronal development. These findings could have implications for understanding the basis of sex-specific responses in the nervous system and their relation to neurological disorders.

## Methods

### Cell lines

The human embryonic stem cell lines WA14 p33 (XY) (Thomson et al., 1998) and WA09 LT2e-H9CAGGFP p80 (XX) (Macarthur et al., 2012) were acquired from WiCell, Madison, Wisconsin. All other lines, HS975 (XX) p16, HS980 (XX) p14, KARO1 (XX) p12, HS1001 (XY) p22, HS983a (XY) p12, HS401 (XY) p33 were acquired from the Karolinska Institute Human Embryonic Stem Cell Bank (Main et al., 2020). The cell lines were tested for common karyotypical abnormalities with the STEMCELL hPSC Genetic Analysis Kit (#07550). None of the lines showed signs of abnormalities (Supplementary Figure S8).

### Cell cultivation and differentiation

The hESC lines were cultivated as monolayers in mTeSR1 plus medium (STEMCELL), on 6-well cell culture dishes (Sarstedt 83.392) coated with Matrigel (Corning). The cultures were maintained in 5% CO_2_ and a temperature of 37°C. Dissociation of the cells was performed using a Versene (ThermoFischer 15040066) incubation of 8 min at room temperature. Each differentiation experiment was conducted in triplicates for each cell line and the protocol was identical for each cell line.

The neural differentiation protocol was based on (Chambers et al., 2009; van de Leemput et al., 2014) and was aimed to create a population of mixed neurons. The initial neural induction was performed on poly-L-ornithine (20 μg/mL, Merck P3655-50 MG) and laminin (20 μg/mL). Each we of a 6-well plate was coated with 1 mL poly-L-ornithine, incubated over night at 8°C followed by an incubation for 1 h at 37°C. Afterwards, the wells were rinsed with cell culture grade H_2_O and coated with 1 mL laminin solution, followed by the same incubation conditions as described before.

Differentiation was induced by dual SMAD inhibition using KO-DMEM/F12 and 1X knockout stem replace (KSR, Thermo Fisher 10828028) together with 10 µM SB-431542 (TargetMol T1726) and 0.1 µM LDN-193189 (TargetMol T1935) for 9 days. On day 3, an additional 3 µM CHIR-99021 (TargetMol T2310) and 1 µM cyclopamine (TargetMol T2825) was added and kept until day 17 of differentiation. On day 5, a transition from KSR medium to a N2 supplement (ThermoFischer 17502048) based medium in DMEM/F12 was performed in 25% steps per day. On day 6 the cells were split 1:3 and reseeded on Matrigel coated plates. On day 18, medium was changed to neurobasal medium with 1X B27 (ThermoFischer 17504044), 55 µM β-mercaptoethanol (ThermoFischer 31350010 µM), 10 µM cAMP (ApexBio B9001), 200 µM ascorbic acid (Merck A4403), 20 ng/mL GDNF (Peprotech 450–150), 20 ng/mL BDNF (Peprotech 450–02) and 50 ng/mL β-NGF (Peprotech 450–01). Each medium also received 2 mM glutamax (ThermoFischer 35050061) and 0.1 mM non-essential amminoacids (ThermoFischer 11140035). Medium was replenished every day until day 17 and afterwards every second day. For the start of the differentiation 150.000 cells were seeded into the coated dishes together with mTeSR1 plus medium. After 24 h of incubation, medium was changed to KSR medium to start the differentiation.

The cell media in this experiment, such as mTeSR+ and NB, as well as their supplements KSR, N2 and B27 can contain hormones such as insulin, progesterone and triiodothyronine. These hormones have proven to be essential for the maintenance and differentiation of stem cells. Except from the presence of progesterone, we have no evidence for the presence of other steroid hormones in the cell cultures.

### RNA sampling and sequencing

RNA sampling for qPCR as well as for RNA sequencing was performed in the following way: cells were washed with 2 mL PBS, thereafter 1 mL TriZol (ThermoFischer 15596026) was used for lysation. The lysate was passed six times through a syringe (BD Microlance 3 (27G ¾ (Nr.20) 0.4 × 19 mm) Ref: 302200) to promote cell lysis. The TriZol phases were separated using chloroform and the RNA phase was purified with the PureLink RNA Mini Kit (ThermoFischer 12183018A). Subsequently, the RNA was depleted of DNA using the DNA-free DNA Removal Kit (ThermoFischer AM 1906). All steps were executed according to manufacturer’s instructions.

Cell lysates were obtained from one 6-well-plate well before differentiation (D0) and after start of differentiation (D4, D9, D17, D27, D37) using 1 mL TriZol (ThermoFischer 15596026). TriZol phases were separated using chloroform and the RNA phase was purified with the PureLink RNA Mini Kit (ThermoFischer 12183018A) according to manufacturer’s instructions. Subsequently, the RNA was depleted of DNA using the DNA-free DNA Removal Kit (ThermoFischer AM 1906).

RNA samples were sent in triplicates for sequencing to the SNP&SEQ Technology Platform in Uppsala where the RNA quality was verified using a Bioanalyzer (RIN ≥8). Samples were then treated with the TruSeq Stranded Total RNA and Ribo-zero Gold kit (Illumina). The treatment preserves strandedness of transcripts but depletes the samples of cytoplasmatic and mitochondrial ribosomal RNA. Sequencing was performed on the NovaSeq instrument with an S4 lane (Illumina) resulting in approximately 27.7 million 150 bp pair-read read-pairs per sample. The data discussed in this publication have been deposited in NCBI’s Gene Expression Omnibus (Edgar et al., 2002) and are accessible through GEO Series accession number GSE249035 (https://www.ncbi.nlm.nih.gov/geo/query/acc.cgi?acc=GSE249035).

### Differential gene expression and gene set enrichment analysis

The samples were aligned to GRCh38_r96 (Schneider et al., 2017) with Hisat2 (Kim et al., 2019) and sequencing reads were assigned to genomic features using featureCounts (Liao et al., 2014). Quality control was performed with FastQC and MultiQC (Ewels et al., 2016). The differential gene expression analysis was performed with DEseq2 (Love et al., 2014). The two factors “Sex” and “Time” were joined and the contrast function was used to compare the differential gene expression between and within the biological sexes (XX to XY) at each timepoint (D0, D4, D9, D37), Figure 3 A-D. Differentially expressed genes were then filtered for a minimal average of 50 counts in any of the cell lines of one sex, and for a fold-change bigger than 0.5 with an adjusted *p*-value lower than 0.05. The count limit of 50 accounts to approximately 2 CPM in our data and is considered stringent. The value was selected after inspection of the histogram of average logCPM values and an appropriate threshold was selected between the low-CPM peak representing non-expressed genes and the high-CPM peak representing expressed genes.

To acquire genes that are truly sex different, a stringent filter was implemented. It consisted of the standard parameters: fold-change (≥0.5) and adjusted *p*-value (≤0.05), but also includes the coefficient of variation for genes within cell lines of each sex (≤0.5) and a minimal difference between the expression of cell lines of the opposite sex (≥20% difference in gene expression). The filter kept only those genes that are expressed at a similar level within cell lines of the same biological sex and if none of the cell lines of one sex expresses the gene of question as high as any cell line of the other sex. For this, the coefficient of variation for genes within the cell lines of each sex was required to be lower than 0.5, to remove genes that are highly variable in expression and hard to predict. Additionally, the difference in gene expression between the cell lines of the opposite sex had to be higher than 20%. The latter required all 9 comparisons (3 male vs. 3 female lines) to display a difference of at least 20% in DEseq2 normalized counts. To investigate genes that contribute significantly to the neuronal differentiation, XX and XY counts were combined and the timepoints were contrasted (D0 to D4, D0 to D9 and D0 to D37), Figures 3E, F.

For the gene set enrichment analysis, the GSEA/MSigDB software was used, software version 4.2.3 and molecular signatures database version 7.5.1 (Mootha et al., 2003; Subramanian et al., 2005). Preranked GSEAs were performed using the stat parameter of the DEseq2 analysis, which represent the difference between the selected comparison including the magnitude of fold-change and p-adjusted. The C5 ontology gene sets were used consisting of gene sets for biological processes (BP), cellular components (CC) and molecular functions (MF). The resulting data was used to create enrichment maps using the Cytoscape software version 3.91 (Shannon et al., 2003) and the auto annotate app for cluster annotation based on gene set description. Enrichment maps were created using an FDR q-value cut-off of below 0.1 and a perfused force directed layout based on the similarity coefficient of gene sets.

### qPCR

For the gene expression analysis by qPCR, cDNA was synthesized from the purified and DNA free RNA using Maxima H Minus Reverse Transcriptase (ThermoFischer EP0752) with anchored Oligo (dT)20 Primer (ThermoFischer 12577011). The following protocol was used for cDNA synthesis in a 15 µL reaction: 500 ng rDNAse-treated RNA, 1.25 μg/μL Oligo (dT)20 Primer, 1 µL dNTP mix (10 mM each, Thermo Fisher R0191), 4 µL RT buffer, 0.5 µL RiboLock RNAse inhibitor (ThermoFisher EO0381), 0.5 µL Maxima RT enzyme and RNAse free water was used to top up to the desired volume. Incubate at 50°C for 30 min, terminate at 85°C for 5 min. A NanoDrop spectrophotometer was used to assess the RNA amount and quality. RNA integrity was measured using gel electrophoresis and comparison of 28S/18S ribosomal RNA ratios. The qPCR analysis was performed on the Applied Biosystems 7500 Real-Time PCR System using PowerUp SYBR Master Mix (Applied Biosystem A25742) and standard cycling mode. For every reaction 1 ng of cDNA was used with 7.5 μL of SYBR master mix and 1 μL of 10 mM forward and reverse primers. Molecular grade H_2_O was added for a total volume of 15 μL. qPCR measurements were taken in triplicates. The thermal cycling profile was selected according to master mix recommendations (1 cycle UDG activation, 50°C, 2 min; 1 cycle activation of Dual-Lock DNA polymerase, 95°C, 2 min; 40 cycles of denaturation, 95°C, 15 s, and annealing/extension, 62°C, 1 min), all runs were performed at an annealing temperature of 62°C. Amplification cycles were repeated 40 times followed by a melting stage, serving as a diagnostic tool to assess amplicon length. Primer sequences can be found in Supplementary Table S9.

## Results

### Similar neuronal differentiation of male and female human embryonic stem cells

**FIGURE 1:**
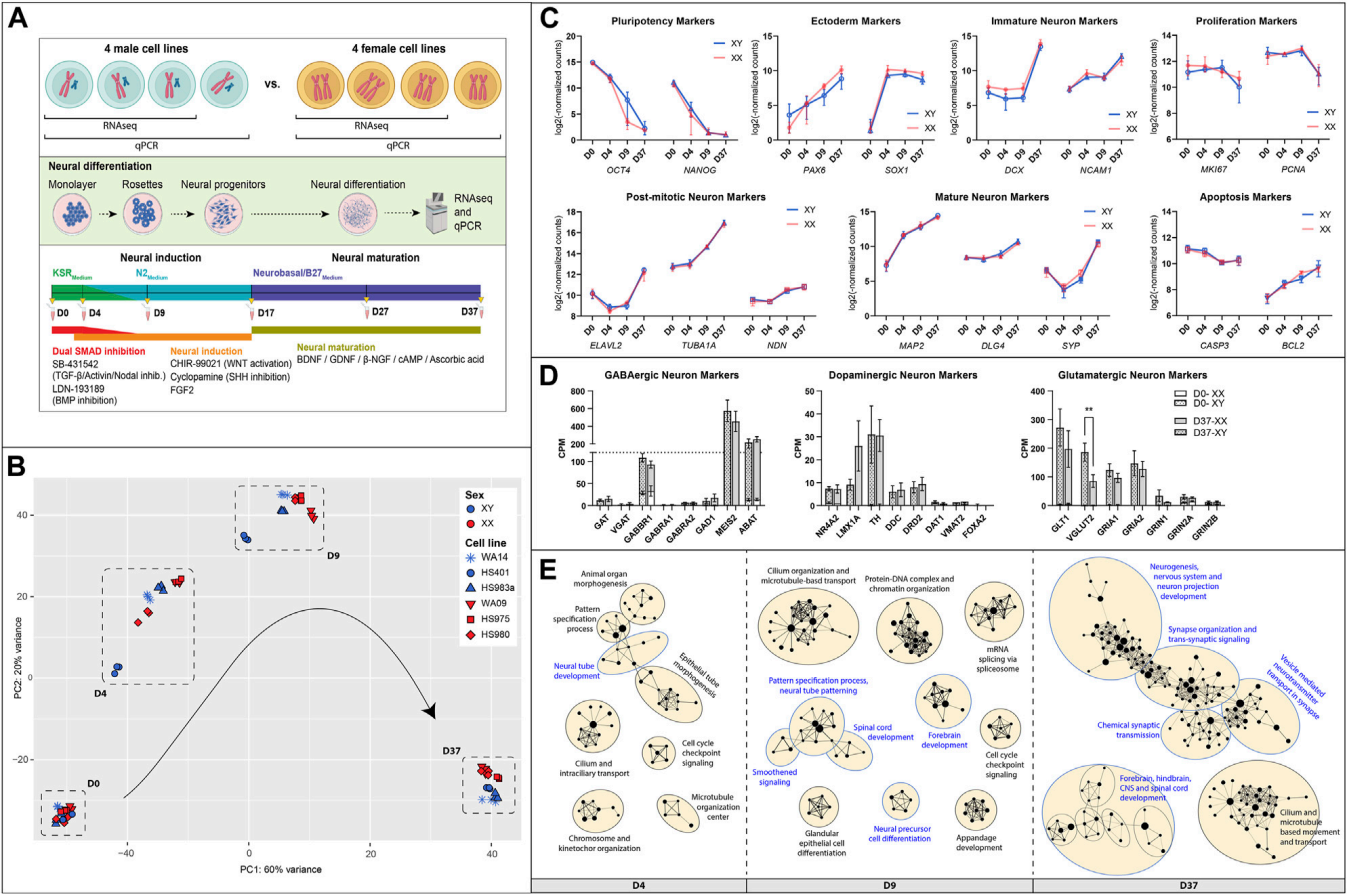
Similarities between male and female hESC in neuronal differentiation: **(A)** Overview of the experimental procedure: Four hESC lines of each sex were differentiated for 37 days to a population of mixed neurons induced by small molecules in 2D culture. Bulk RNA sequencing and qPCR. **(B)** A PCA analysis of expressed genes displays similar gene expression patterns at D0, some inter-cell line differences during early differentiation, and a sex-biased separation at D37 (XY: WA14, HS401, HS983a, XX: WA09, HS975, HS980). **(C)** Marker gene expression for pluripotency (OCT4, NANOG), ectoderm (PAX6, SOX1), immature neurons (DCX, NCAM), mature neurons (MAP2, DLG4, SYP), post-mitotic neurons (ELAVL2, TUBA1A, NDN), as well as for proliferation (MKI67, PCNA), and apoptosis (CASP3, BCL2) show a similar expression in male and female cell lines. **(D)** Markers for specialized neurons (GABAergic, Dopaminergic, Glutamatergic) display a premature state of the neurons after 37 days of differentiation. **(E)** A gene set enrichment analysis at D4, D9, and D37 reveals an upregulation of biological processes implicated in neuronal differentiation in all cell lines. The GSEA was summarized in an enrichment map. Each dot represents a gene set; the size of the dot corresponds to the size of the gene set. If the dots are connected or in close proximity, the more the gene sets are overlapping, and the closer related are their associated biological functions. Gene sets were grouped by a common GO-term classification and named after an overarching biological function. Biological functions implicated in neuronal development are highlighted in blue. Gene sets are filtered by FDR q-values <0.1. Figure 1A created with BioRender.com.

To identify potential sex differences in the development of human embryonic stem cells (hESC), we differentiated four male and four female-derived hESC lines towards a population of mixed neurons. The differentiation protocol utilizes adherent cell culture combined with small molecules for neural induction and maturation, as described in detail in the Methods section. The cells were differentiated for a total of 37 days, and samples were collected at six timepoints: D0, D4, D9, D17, D27, and D37 (Figure 1A). Six cell lines were analyzed by total RNA sequencing at four timepoints (D0, D4, D9, D37), and all 8 cell lines were analyzed via qPCR at all six timepoints (Table 1).

**TABLE 1:** hESC lines and gene expression analysis method and timepoints.

Karyotype 22 + XY	Karyotype 22 + XX	Gene expression analysis method	Timepoints RNAseq	Timepoints qPCR
WA14	WA09	RNAseq + qPCR	D0, D4, D9, D37	D0, D4, D9, D17, D27, D37
HS-980a	HS-980	RNAseq + qPCR	D0, D4, D9, D37	D0, D4, D9, D17, D27, D37
HS-401	HS-975	RNAseq + qPCR	D0, D4, D9, D37	D0, D4, D9, D17, D27, D37
HS-1001	KARO1	qPCR	-	D0, D4, D9, D17, D27, D37

A principal component analysis of expressed genes revealed that male and female cell lines show similar gene expression at D0 but display cell line-specific differences at D4 and D9 (Figure 1B). After 37 days of differentiation, the differences decreased, and male as well as female cell lines show similar gene expression. However, we noticed a clear sex-specific separation between them according to principal component 2 (PC2).

In general, the cell lines of both sexes have differentiated similarly towards neurons, displayed by very similar expression dynamics of marker genes for pluripotency (*OCT4*, *NANOG*), ectoderm (*PAX6*, *SOX1*), immature neurons (*DCX*, *NCAM1*), mature neurons (*MAP2*, *DLG4*, *SYP*), post-mitotic neurons (*ELAVL2*, *TUBA1A*, *NDN*), as well as for proliferation (*MKI67*, *PCNA*) and apoptosis (*CASP3*, *BCL2*), Figure 1C. In addition, the pluripotency state of all cell lines was investigated and identified as primed by the overexpression of naive over primed pluripotency marker genes at T0, Supplementary Figure S1. To estimate the replicability of the differentiation protocol, the experiment was repeated two more times, and the expression of *NES*, *PAX6,* and *DCX* was measured using qPCR. We observed consistent gene expression levels in each repetition, indicating low variability and a robust differentiation protocol. Additionally, we identified sex differences which were not detected in the RNAseq data for *NES* at D9 (1.8 FC, *p* < 0.0001), in PAX6 at D37 (1.3 FC, *p* < 0.05), and in *DCX* at D17 (2.2 FC, *p* < 0.0001), Supplementary Figure S2.

An investigation of marker genes for specialized neurons has revealed that male as well as female cells similarly differentiated to premature neuron-like cells after 37 days (Figure 1D). For example, the premature state of GABAergic neurons is suggested by the high expression of *GABBR1* (GABA type B receptor unit) combined with high expression levels of *MEIS2* and *ABAT* (both GABAergic precursor markers), while other GABAergic markers, such as the type A receptor *GABRA1/2* or the neurotransmitter transporters *SLC6A1* (*GAT*) and *SLC32A1* (*VGAT*), as well as the rate-limiting enzyme for GABA synthesis *GAD1* are expressed very low or not at all. Similarly, dopaminergic markers *NR4A2* and *LMX1A*, which are transcriptional regulators for the development of DA neurons, indicate the presence of neurons that are not fully mature. However, the expression of the catecholamine precursor L-DOPA-synthesizing-enzyme tyrosine hydroxylase (*TH*) and the L-DOPA to dopamine converting enzyme dopa decarboxylase (*DDC*), as well as the dopamine receptor D2 (*DRD2*), point to an, at least, early post-mitotic state of some dopaminergic neurons**.** The dopamine transporters *SLC6A3* (*DAT1*) and *SLC18A2* (*VMAT2*), as well as the transcription factor *FOXA2* that supports maintenance of mature DA neurons, are expressed at low levels or not at all. As for the glutamatergic neurons, we can see high expression of the glutamate transporters *GLT1* and *VGLUT2,* as well as for the glutamate AMPA type receptors subunits *GRIA1* and *GRIA2*. Glutamate NMDA type receptors were also expressed, albeit at a moderate to low level (data not shown). Interestingly, the *VGLUT2* gene shows a sex difference and is higher expressed in male cells than in female cells after 37 days of differentiation (*p* = 0.009 **).

Gene set enrichment analyses (GSEA) also revealed a similar differentiation of male and female cell lines. We performed GSEAs for each sex separately (e.g., XY D0 to D4 and XX D0 to D4) and compared them. We found that the upregulated gene sets representing biological processes in the cells during the differentiation experiment are almost identical with only minor variations between the sexes (data not shown). A simple way to visualize this is by performing a GSEA with pooled data from all cell lines. This has resulted in a single enrichment map in which gene sets will only appear if they are upregulated in all cell lines, male and female (Figure 1E). Each dot in the map represents an upregulated gene set. Upregulated processes involved in neuronal development are, e.g., neural tube development at D4, forebrain development at D9, and neurogenesis, nervous system, and neuron projection development at D37. The processes involved in nervous system development are highlighted in blue. A full list of the enriched gene sets can be found in Supplementary Table S1.

### Differences in XCI erosion in female cell lines displays little effect on autosomal and X-linked gene expression

Since X chromosome inactivation (XCI) erosion can affect the gene expression of both X-linked and autosomal genes, we investigated the XCI erosion status in our female cell lines. A key indicator for the presence of XCI erosion is the absence of XIST expression. In our study, one of the three primed female cell lines used for RNAseq analysis expresses XIST at a relatively low level (HS980 with 1.5 TPM at D0) without significant changes during differentiation. The other 2 cell lines (WA09/LT2e and HS975) do not express XIST at any time point. To assess the extent of XCI erosion in the female cell lines, we calculated the expression ratio of X-linked genes between male and female cell lines. Since XCI erosion leads to an increased expression of X-linked genes, we can use the XX:XY expression ratio to estimate the degree of XCI erosion. In line with Bar et al., 2019, we consider a ratio of >1.1 as a state in which X-linked genes in female cell lines are increasingly expressed due to XCI erosion. XCI escape genes display an average XX:XY ratio of 1.29 (data not shown) and were removed before the analysis.

On average, the majority of X-linked genes (67%) in the three female lines demonstrate an expression ratio indicative of one X chromosome being inactivated (XaXi), while the remaining 33% of X-linked genes reflect either a state of XCI erosion (XaXe) or two active X chromosomes (XaXa), Supplementary Figure S3A. When analyzed individually, the cell lines exhibit different states of XCI erosion. The lines HS975 and WA09/LT2e display a higher degree of XCI erosion with 31% and 48% of X-linked genes, respectively, with an XX:XY ratio above 1.1 and thus within the XaXe or XaXa range. While in the line HS980 only 16% of X-linked genes display a ratio above 1.1 (Supplementary Figure S3B). To estimate the number of affected genes, we used the average XX:XY gene expression ratio of chromosome 5 and 16 as a baseline, given their similarity in the number of protein-coding genes compared to chromosome X. After subtracting the baseline, the final fraction of X-linked genes upregulated due to the potential effect of XCI erosion was estimated to be on average 6%, corresponding to 38 out of 634 genes (4% or 25 genes for HS980, 4.5% or 29 genes for HS975% and 10% or 63 genes for WA09/LT2e). The complete list of X-linked genes and their corresponding XX:XY expression ratios can be found in Supplementary Table S2. Based on this, only two of the gametologous genes (*CASK* and *SHROOM2*) were identified as possibly affected by XCI erosion since they exhibit an XX:XY ratio above 1.1 and are not XCI escape genes. Furthermore, no significant difference was found between the average X-linked gene expression of the cell lines using a one-way ANOVA with Tukey’s multiple comparison test, (Supplementary Figure S3C). Moreover, the variance of the average gene expression from the X-chromosome among the male cell lines (F = 1.33) was twice as high than among the female cell lines (F = 0.65). This suggests that the variation in *XIST* expression does not critically impact X chromosome gene expression levels in the female lines, rendering them comparable in their X-linked gene expression despite their difference in *XIST* expression and XCI erosion.

To evaluate whether *XIST* expression and XCI erosion status affect the autosomal gene expression in the female cell lines, we investigated the relation between X chromosomal and autosomal gene expression. For this, we used the TPM gene expression values to calculate the X:A (Supplementary Figure S3D). We did not observe significant difference in autosomal gene expression in the Xist-positive cell line HS980. A slight trend for increased expression can be noted on chromosomes 8, 9, 11, and 22 with their ratios increased by 0.15–0.2. However, the average X:A ratio of all autosomes was only slightly increased by 0.09 points in HS980, compared to the average of the Xist-negative lines (HS980: 1.16, LT2e: 1.11, HS975: 1.03).

### X and Y-linked genes contribute to neuronal differentiation

**FIGURE 2:**
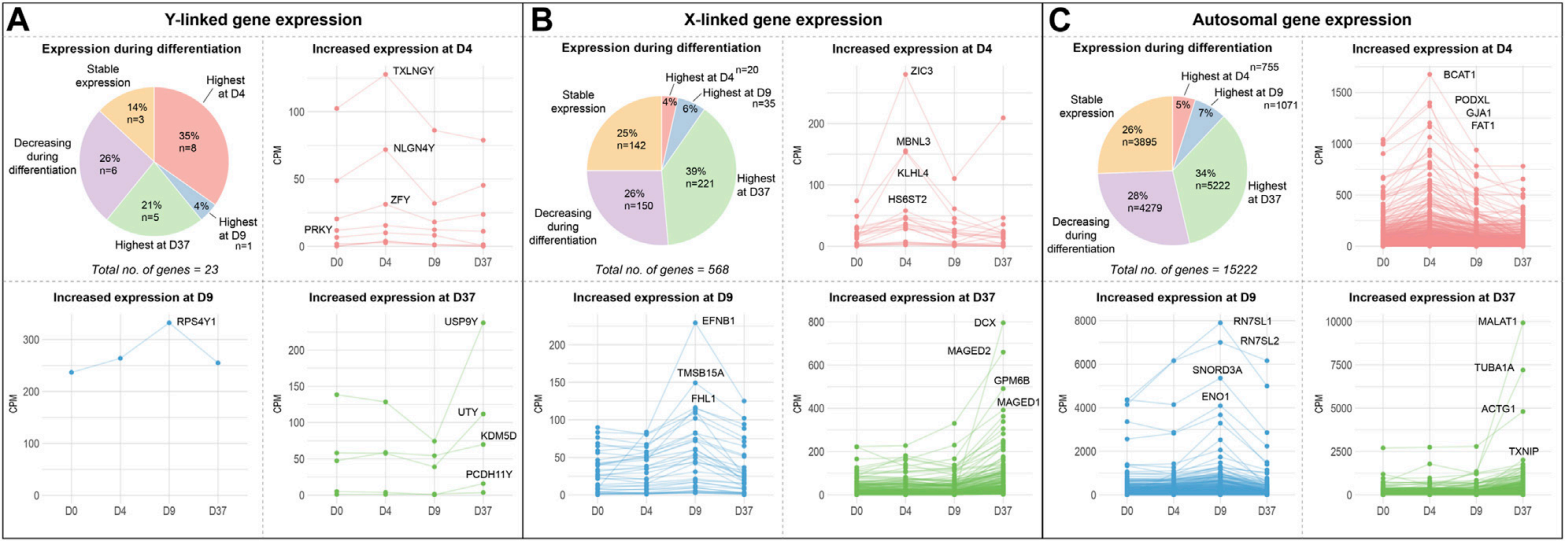
Gene expression of sex chromosome and autosomal genes during differentiation: Gene expression of **(A)** Y-linked, **(B)** X-linked, and **(C)** autosomal gene expression during neural differentiation. The pie-chart shows the number of genes peaking in expression at different timepoints (D4, D9, or D37), as well as the number of genes decreasing during differentiation and genes that maintained a stable expression level. Additional panels display the expression dynamics of genes peaking at D4, D9, and D37. The genes with the highest expression levels at the corresponding timepoint are labeled. According to their expression peaks, Y-linked genes play a role at different timepoints during neural differentiation in male stem cell lines.

To identify how many Y-linked, X-linked, and autosomal genes contribute to neuronal differentiation, we investigated their gene expression levels and changes during the differentiation experiment (Figures 2A–C; Supplementary Table S3). We used the average gene expression value of all cell lines grouped by sex and implemented a rigorous threshold by excluding genes with a count number below 50. This stringent criterion was applied to effectively remove a large number of lowly expressed genes. Subsequently, we categorized resulting genes based on the timepoint at which they are expressed highest. We noticed a total of 15,222 autosomal genes, 568 X-linked genes and 23 Y-linked genes to be expressed in the embryonic stem cell lines (Supplementary Table S3). For autosomal and X-linked genes, a similar proportion of genes were upregulated at certain timepoints during differentiation. The largest share of genes was upregulated at D37, accounting for up to 34% of autosomal genes and 39% of the X-linked genes. This is followed by genes that were downregulated during differentiation (26% autosomal, 28% X-linked) and, again in a similar proportion, genes with unchanged expression during differentiation (25% autosomal, 26% X-linked). Only a small percentage of X-linked and autosomal genes seemed to be upregulated at the stages of neuronal induction at D4 (4%–5%) and at D9 (6%–7%). As for the Y-linked genes, the largest number of genes was upregulated early during neuronal induction (35%, 8 genes) at D4. Only one gene is upregulated at D9 in the late stage of neuronal induction (4%, 1 gene).

An equal number of Y-linked genes are upregulated at D37 (21%, 5 genes) or decreased during neuronal differentiation (26%, 6 genes), while 14% (3 genes) of Y-linked genes do not change their expression during differentiation. We have labeled the four genes with the highest expression during differentiation at their corresponding timepoints in the panels D4, D9, and D37 in Figure 2. The Y-linked genes with the highest expression were *TXLNGY*, *NLGN4Y,* and *ZFY* at D4, *RPS4Y1* at D9, and *USP9Y*, *UTY*, *KDM5D*, and *PCDH11Y* at D37 (Figure 2A). The X-linked genes with the highest expression were *ZIC3*, *MBNL3,* and *KLHL4* at D4, *EFNB1*, *TMSB15A,* and FHL1 at D9, and *DCX*, *MAGED2*, *GPM6B,* and *MAGED1* at D37 (Figure 2B). The autosomal genes with the highest expression were *BCAT1*, *PDOXL*, *GJA1,* and *FAT1* at D4, *RN7SL1*, *RN7SL2*, *SNORD3A,* and *ENO1* at D9, and *MALAT1*, *TUBA1A*, *ACTG1,* and *TXNIP* at D37 (Figure 2C). The expression data of these and additional genes, as well as genes that significantly decrease during differentiation or remain stable in expression, can be found in Supplementary Table S3 and Supplementary Figure S4.

### Identifying genes with sex-biased expression and impact on neuronal differentiation

To identify sex-biased gene expression during neuronal differentiation, we performed a differential gene expression analysis with the total RNA sequencing (RNAseq) data. In the assay, we compared 3 male and 3 female cell lines at 4 time points during differentiation, this resulted in the following four comparisons XX to XY at D0, D4, D9, and D37. After applying a filter to exclude genes with low expression (<50 counts), and an adj. *p*-value >0.05, we obtained 1,152, 2,330, 1,497, and 2,915 differentially expressed genes (DEG) for D0, D4, D9, and D37, respectively (Table 2). However, many of those genes did not meet our requirements for sex-based differential expression. Some DEG displayed a too high variation in expression between the cell lines of one sex to be a reliable candidate for a gene with a sex-biased expression. In other cases, individual cell lines of one sex showed a too similar expression level to cell lines of the opposite sex, so that also these candidates were not a good match for a gene with a sex-bias in expression. To ensure that we select true sex differences, we applied another set of stringent *post hoc* filtering rules that only assign genes as sex-biased if they are expressed at a similar level within cell lines of the same biological sex (coefficient of variation <0.5), and that required all cell lines of one sex to express a gene at least 20% higher (based on DEseq2 normalized counts) than all cell lines of the other sex. Additional details can be found in the methods section. After filtering, the numbers of differentially expressed genes have reduced substantially to 65, 218, 114, and 148 for D0, D4, D9, and D37, respectively (Table 2). The complete list of DEG can be found in Supplementary Table S4.

**TABLE 2:** Number of differentially expressed genes between biological sexes during neuronal differentiation.

Timepoint	D0		D4		D9		D37	
**DEG**	1.152		2.33		1.467		2.915	
**Sex-biased DEG** (after filtering)	65		218		114		148	
Sex	Male	Female	Male	Female	Male	Female	Male	Female
**DEG on chromosome Y**	16	-	17	-	14	-	15	-
**DEG on chromosome**	-	21	-	29	-	17	-	21
**X** (known XCI-escapees*)		*(11)*		*(12)*		*(10)*		*(9)*
**Autosomal DEG**	11	17	119	52	14	69	89	23
**Total DEG**	27	38	137	81	28	86	104	44

^*^XCI-escaping genes selected according to Katsir and Linali (2019), Garieri et al. (2018), Zhang et al. (2013).

**FIGURE 3:**
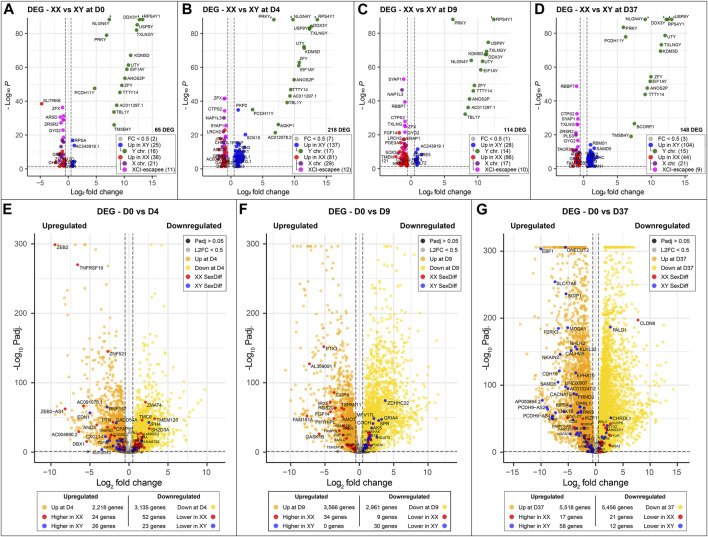
Sex-specific differences at key timepoints and in genes that change significantly during differentiation: **(A–D)** Sexually differentially expressed genes comparing male (XY) against female (XX) cell lines at different time points of neuronal differentiation. Genes overexpressed in male cell lines are marked in blue; green if they are located on the Y chromosome. Similarly, genes overexpressed in female cell lines are marked in red; purple if they are expressed from the X chromosome and pink if they escape XCI. The number of sex-biased genes overexpressed in male and female cell lines is as follows: **(A)** D0, 65 genes in total, 25 in XY and 38 in XX; **(B)** 218 genes in total, 137 in XY and 81 in XX; **(C)** 114 genes in total, 28 in XY and 86 in XX; **(D)** 148 genes in total, 104 in XY and 44 in XX. The number of genes with sex-biased expression from sex chromosomes remains relatively constant with an average of 15 Y chromosome and 22 X chromosome genes expressed at each time point. XCI-escapee genes are among the sex-biased genes with the highest *p*-value in the XX cell lines. **(E, F)** display the DEG that are up- and downregulated over the course of neuronal differentiation. Genes that are additionally expressed in a sex-biased manner are highlighted in red and blue according to their sex-bias in female and male cell lines, respectively. On average, 3,500 genes are up- and downregulated at each time point, of which 1% display sex-biased expression. **(E)** From D0 to D4: 2,218 genes are upregulated (26 in XY and 24 in XX with a sex-bias) and 3,135 are downregulated (23 in XY and 52 in XX with a sex-bias); **(F)** From D0 to D9: 3,566 genes are upregulated (0 in XY and 34 in XX with a sex-bias) and 2,961 are downregulated (30 in XY and 9 in XX with a sex-bias); **(G)** From D0 to D37: 5,518 are upregulated (58 in XY and 17 in XX with a sex-bias) and 5,456 are downregulated (12 in XY and 21 in XX with a sex-bias).

As expected, genes of the sex chromosomes (X and Y) make up a considerable part of the sex-biased DEG at all time points (Table 2). The Y chromosome genes display the highest fold-changes among the DEG in male cells. Similarly, the X chromosome genes make up a large part of the overexpressed genes in female cells. Not surprisingly, the X-linked genes that escape X-inactivation (XCI-escapees) show the highest fold-change among the DEG in female cells. Apart from the genes on the sex chromosomes, there is also a significant share of DEG expressed from the autosomes, 43% at D0, 78% at D4, 73% at D9, and 76% at D37. Interestingly, the number of sex-biased DEG increases with the start of differentiation at D4 (Figures 3A–D). While at D0 there are 65 sex-biased genes expressed (27 XY, 38 XX), at D4 it is 218 genes (137 XY, 81 XX), at D9 it is 114 genes (28 XY, 86 XX), and at D37 it is 148 genes (104 XY, 44 XX). Next, we investigated the sex differences at D37 in more detail. Most of the sex-biased genes at D37 are higher expressed in the male cell lines, 104 compared to 44 genes in the female lines (Figure 3D; Supplementary Table S4). From the upregulated genes in the female lines, 21 are X-linked genes, and of these, 9 are known to escape XCI, the remaining 22 genes are autosomal. In contrast, the largest number of upregulated genes in males are expressed from the autosomes (89 genes). Only a small number of the upregulated genes at D37 in the male lines are encoded on the Y chromosome (15 genes). We have annotated all sex-biased genes at D37 for their appearance in neuronal, synaptic, as well as developmental gene sets using the gene ontology database (GO) and collected evidence from available scientific literature regarding their implication in neuronal processes (Supplementary Table S5). Of the 104 DEG in male cell lines, 32 genes were implicated in neuronal processes (Top 5: *NLGN4Y*, *UTY*, *PCDH11Y*, *NHLH2*, *EBF1*), while of the 43 DEG in female cell lines it was 7 genes (Top 5: *SYAP1*, *TACR3*, *GRM1*, *NDNF*, *AMOT*). Genes that change their expression pattern during neuronal differentiation and do that in a sex-dependent manner are likely to be important candidates that cause sex differences in brain development. To identify these genes, we looked for sex-biased genes among the highly up- and downregulated genes during differentiation (D0 to D4, D0 to D9, and D0 to D37) and found between 2,218 and 5,518 genes that are up- or downregulated, respectively (Figures 3E–G). Approximately 1% of the genes show a sex-bias in gene expression (Supplementary Table S6). We have added this sex-biased expression during neural differentiation as a parameter in Supplementary Table S5 and used it for the selection of candidate genes later.

### Sex-biased gene expression in neural processes after 37 days of differentiation

**FIGURE 4:**
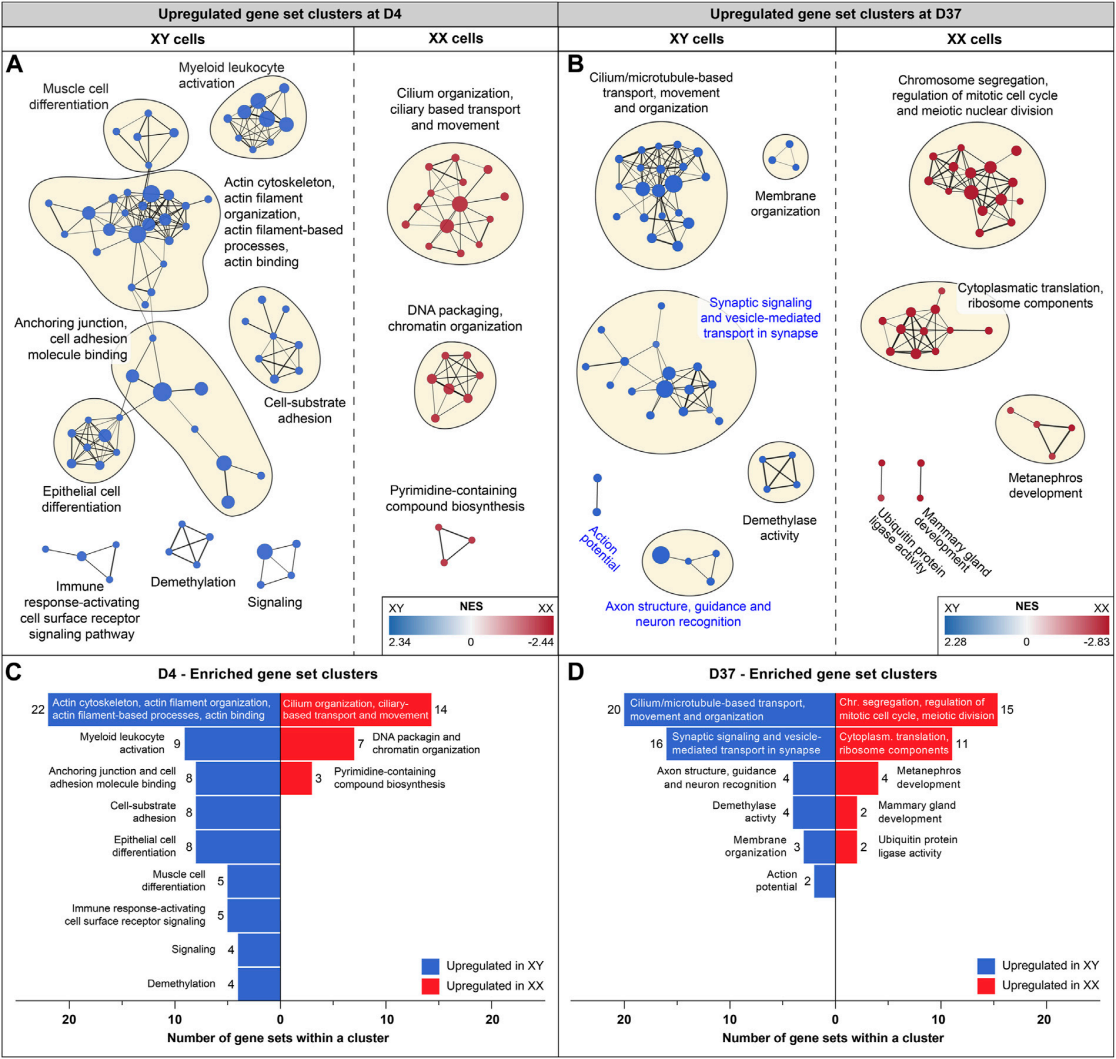
Enriched gene sets at D4 and D37: Gene set enrichment analysis was based on differentially expressed genes at D4 and D37. Enriched gene sets in male and female cell lines are colored in blue and red, respectively. Panels **(A, B)** display an enrichment map in which a dot represents an upregulated gene set. If gene sets are connected, and the closer they are in proximity, the more they are related in function. Clusters of gene sets are encircled and named after their common biological function. **(A)** Enrichment map for D4: male cell lines display upregulated gene sets that are associated with actin-based processes, such as “Cell-substrate adhesion”, “Anchoring junction” and “Actin cytoskeleton and actin filament organization”. Female cell lines at D4 display larger gene set clusters of cilium-based processes as well as “DNA packaging and chromatin organization”. **(B)** Enrichment map for D37: male cell lines show three clusters of enriched gene sets that are associated with neuronal processes (cluster names highlighted in blue) and a larger cluster that combines cilium and microtubule-based processes. Female cell lines at D37 show an upregulation of gene sets involved in cell division and ribosomal processes. Subfigures **(C)** and **(D)** summarize upregulated gene sets in male and female cell lines (at D4 and D37) and display the number of gene sets within a cluster.

To identify potential differences in biological processes between cell lines of different sexes, we conducted additional GSEAs comparing male and female gene expression at the timepoints D0, D4, D9, and D37. Enrichment maps were generated from the GSEA results to identify clusters of upregulated gene sets with similar functions. Clusters of gene sets are more likely to have a biological relevance than isolated gene sets. Using this approach, identified various biological processes before differentiation and at the early (D4) and late stages of differentiation (D37). Interestingly, at D9, the analysis did not reveal any differences between the cell lines. Before differentiation (D0), upregulated gene set clusters in male cell lines were associated with ribosomal and mitochondrial functions (Supplementary Figure S5). At D4, a substantial number of actin-based processes were increased in male cells, while in female cells, cilium-associated processes, as well as DNA packaging and chromatin organization, were upregulated (Figures 4A, C). At D37, male cells exhibited upregulation in cilium and microtubule-based processes, as well as in various neural processes such as synaptic signaling, synaptic vesicle transport, axon structure, axon guidance, neuron recognition, and action potential. In contrast, female cell lines were upregulated in cell division and ribosomal processes (Figures 4B, D). Details for all enriched gene sets within a cluster can be found in Supplementary Table S7.

**FIGURE 5:**
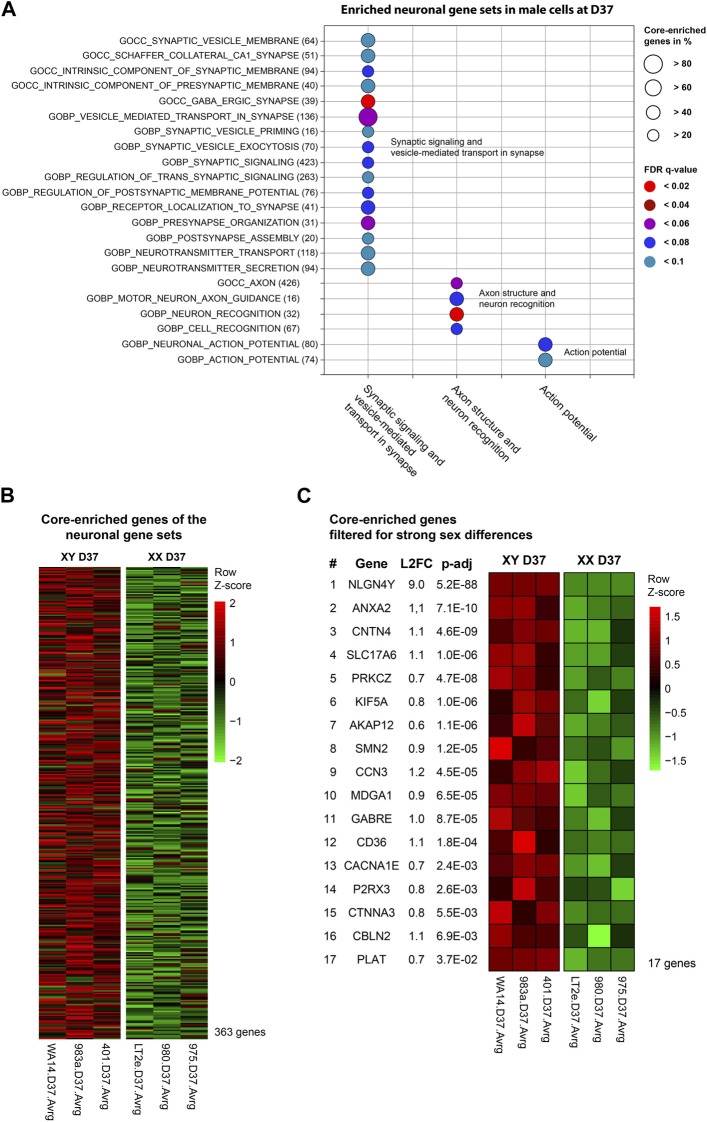
Enriched neuronal gene sets and corresponding core-enriched genes in male cell lines at D37: Core-enriched genes from enriched neuronal gene sets in male lines at D37 were contributing to candidate gene selection. **(A)** Gene sets upregulated within the neuronal clusters. Numbers in brackets show the total number of genes within the gene set. Circle size illustrates the percentage of core-enriched (highly contributing) genes within the gene sets. Color indicates the false-discovery rate (q-value) of the gene set at comparison of male and female cell lines. **(B)** Gene expression heatmap of core-enriched genes from all gene sets within the sex-biased neural process clusters. Colors indicate the level of expression comparing male and female cell lines based on a Z-score. Red indicates an overexpression, black indicates an equal expression in both cell lines, green indicates a reduced expression. **(C)** Using stringent filtering, we identified 17 core-enriched and significantly sex-biased genes within the clusters. These genes later contributed to the selection of candidate genes.

At D37, the GSEA identified three clusters of sex-biased neural processes in male cell lines. To pinpoint the specific genes contributing to these clusters, consisting of 22 individual gene sets, we extracted the genes with the highest contribution to the gene set enrichment, known as core-enriched genes (Figure 5A). This resulted in 362 unique core-enriched genes (Figure 5B), which we further filtered using previously mentioned stringent filtering rules, to identify 17 sex-biased genes implicated in neural processes (Figure 5C **)**. The full list of core-enriched genes is available in Supplementary Table S8, with the 17 sex-biased genes highlighted.

### Combined information returns candidate genes that show robust sex-biased overexpression in the cell lines and are implicated in neural processes

**FIGURE 6:**
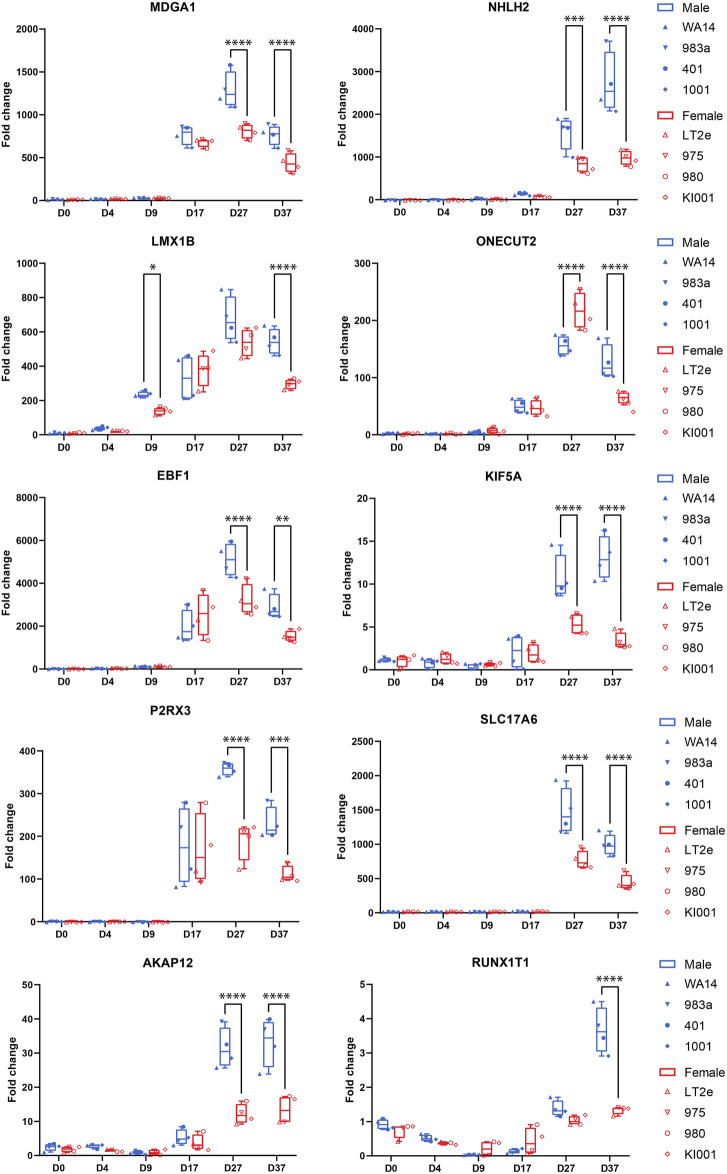
Candidate gene expression at all time points: Gene expression was measured by qPCR in replicates of the neural differentiation experiment (*n* = 3). All candidate genes (*MDGA1, NHLH2, LMX1B, ONECUT2, EBF1, KIF5A, P2RX3, AKAP12, SLC17A6, RUNX1T1*) show significant overexpression in all four male cell lines at D37. Many genes start expressing at D17. RUNX1T1 shows a significant sex difference already at D9. ONECUT2 shows a female-biased sex difference at D27, which then changes to an overexpression in male cells at D37. Differences between the male and female samples were measured using repeated measurements two-way ANOVA with Šidák’s multiple comparisons test.

Next, we aimed to identify candidate genes demonstrating robust sex differences in expression and implication in the development and function of neurons. For this, we combined all the information gathered in this study to annotate the sex-biased genes that resulted from the DEG analysis at D37 (104 upregulated genes in XY cells and 44 in XX cells). The following information was used for the annotation: (a) GO-terms associated with neurodevelopment, (b) upregulation at D37 of differentiation, (c) appearance in clusters of neuronal processes in the GSEA, (d) indications from the literature for involvement in neurodevelopmental processes, and (e) the 17 sex-biased genes resulting from the GSEA analysis of D37. Based on these annotations more than 60 of the 148 genes display implications in neuronal development. Among these genes, 13 displayed an expression level above the 70th percentile, which we decided to select as candidate genes. Ten of these genes display an upregulated gene expression in male cell lines (*NHLH2*, *EBF1*, *SLC17A6*, *RUNX1T1*, *KIF5A*, *AKAP12*, *MDGA1*, *ONECUT2*, *P2RX3*, *LMX1B*), and 3 genes are upregulated in female cell lines (*SYAP1*, *AMOT*, *PAK3*), as detailed in Table 3. The full list of annotated genes can be found in Supplementary Table S5. To validate the expression tendencies of the candidate genes, we conducted qPCR analyses on material from a replicate differentiation experiment, including two additional cell lines for each sex (KARO1-XX and HS1001-XY) and two extra timepoints, D17 and D27. By including timepoints D17 and D27, we observed that most candidate genes display an initial expression earliest at D17 (Figure 6). Considering this and the onset of *DCX* expression at D17 (Supplementary Figure S2), we regard D17 as the earliest timepoint where neurons begin to emerge in our differentiation experiment. The sex-biased expression of candidate genes was affirmed at D37 in all cell lines, with some genes even exhibiting a sex difference as early as D27. The difference between the male and female samples was tested using a repeated measurements two-way ANOVA with Šidák’s multiple comparisons test.

**TABLE 3:** Description of candidate genes: Sex-biased genes overexpressed in male (pos. Log2FC) or female cell lines (neg. Log2FC) with implications in neuron development.

Gene	Log2 FC	p-adj	Description
Overexpressed in male cell lines			
*NHLH2*	1	1.90E-11	Neuronal transcription factor, highly involved in KISS1 neuron development. Implicated in onset of puberty through regulation of kisspeptin and neurokinin-b, as well as GnRH-regulating neurons
*EBF1*	1	3.34E-10	Transcription factor implicated in Cajal-Rezius neurons, telencephalic progenitor cells, and general neural development, especially medium spiny neurons
*SLC17A6*	1.1	1.03E-08	Transporter molecule for glutamate and other neurotransmitters. Especially expressed in dopaminergic neurons where it serves a neuroprotective role
*RUNX1T1*	1	4.84E-07	Neuronal transcription factor implicated in neurogenesis and neuron differentiation
*KIF5A*	0.8	9.99E-07	Microtubule-dependent motor protein essential for the transport of organelles, proteins, and RNA. In neurons, they are required for slow axonal transport of, e.g., neurofilaments and transport within mitochondria. KIF5s are critical for the structural assembly of neurons, maintenance, and dendritic branching
*AKAP12*	0.6	1.12E-06	Kinase anchoring protein. Scaffold protein in synaptic signal transduction. Activation of cAMP-dependent PKA and signaling by Rho GTPases. Essential for synaptic long-term potentiation, synaptic transmission, and synaptic plasticity
*MDGA1*	0.9	6.50E-05	MDGAs are GPI-anchored cell surface glycoproteins. They anchor, e.g., acetylcholesterinase and alkaline phosphatases to defined locations. MDGAs are predominantly expressed in the developing CNS and have a role in cell adhesion, migration, axon guidance during brain development and neuronal migration
*ONECUT2*	0.7	1.17E-03	Transcription factor regulating development of photoreceptor cells. Implicated in 5-HT neuron development and early-born interneurons, spinal cord, enteric, and motor neurons
*P2RX3*	0.8	2.62E-03	Purinergic receptor, extracellular ATP-triggered ion channel. Implicated in sensory and autonomic neuron development, as well as spiral ganglion neuron branching
*LMX1B*	1.2	1.83E-02	Transcription factor, homeodomain with two zinc-binding domains. Implicated in glutamatergic, dopaminergic, and serotonergic neuron development and survival
Overexpressed in female cell lines			
*SYAP1*	−0.8	4.12E-30	Synapse-associated protein. Unclear function in humans but associated with mental retardation, developmental delay, and autism spectrum disorder. In mice, Syap1 is widely expressed in the central nervous system, specifically in axons and growth cones
*AMOT*	−0.7	5.19E-05	Angiomotin, regulator of tight junctions organizing contact points between adjacent cells. Amot has critical roles in neural stem cell differentiation, dendritic patterning, and synaptic maturation
*PAK3*	−0.6	1.90E-03	PAK serine/threonine protein kinase 3 expression is restricted to the brain. Loss-of-function in PAK3 is associated with X-linked intellectual disability. Knockouts lead to reduced neuronal dendrites and brain size. PAK3 regulates spine morphology and number, and neurite complexity

### Gene dosage compensation of X/Y homologs escaping XCI through Y homolog and significant Y homolog overexpression in *TXLNG*/*Y* and *KDM6A*/*UTY*

**FIGURE 7:**
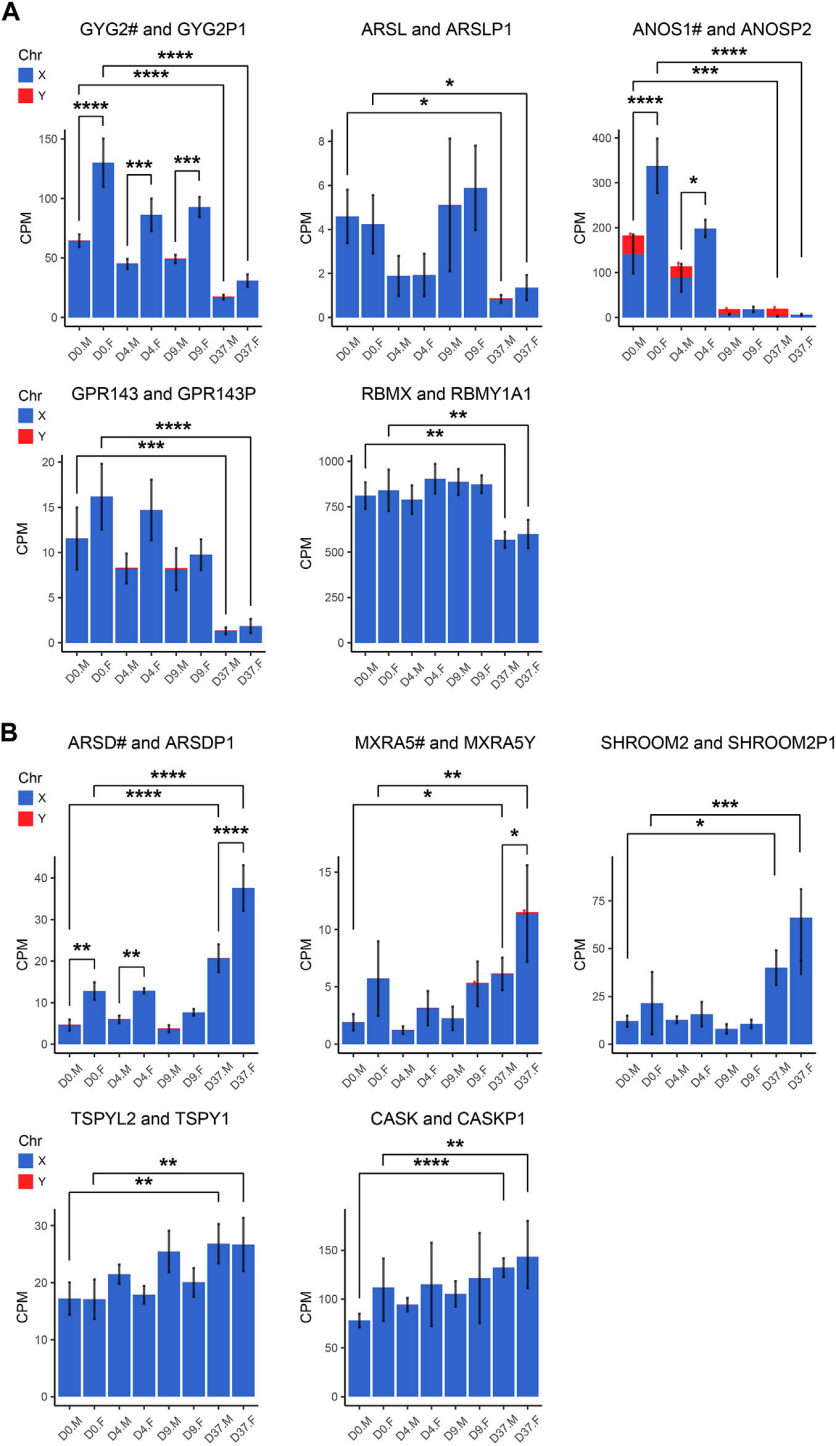
Gametologous genes with low Y homolog expression: X/Y homolog expression was analyzed in total RNA sequencing data (3 male and 3 female cell lines). A hashtag after the X homolog indicates that the gene is escaping XCI. Panel **(A)** displays the 5 gene pairs that decrease (GYG2/P1, ARSL/P1, ANOS1/P2, GPR143/P, RBMX/Y1A1), and **(B)** the 5 gene pairs that increase (ARDS/P1, MXRA5/Y, SHROOM2/P1, TSPYL2/TSPY, CASK/P1) in expression during neural differentiation, comparing the period D0 to D37. An increased gene dosage in females was found in GYG2/GYG2P1 at D0, D4, and D9 (*p* < 0.0001, *p* < 0.001, *p* < 0.001, respectively) and ANOS1 at D0 and D4 (*p* < 0.001, *p* < 0.05). Differences between the male and female samples were measured using repeated measurements two-way ANOVA with Šidák’s multiple comparisons test.

**FIGURE 8:**
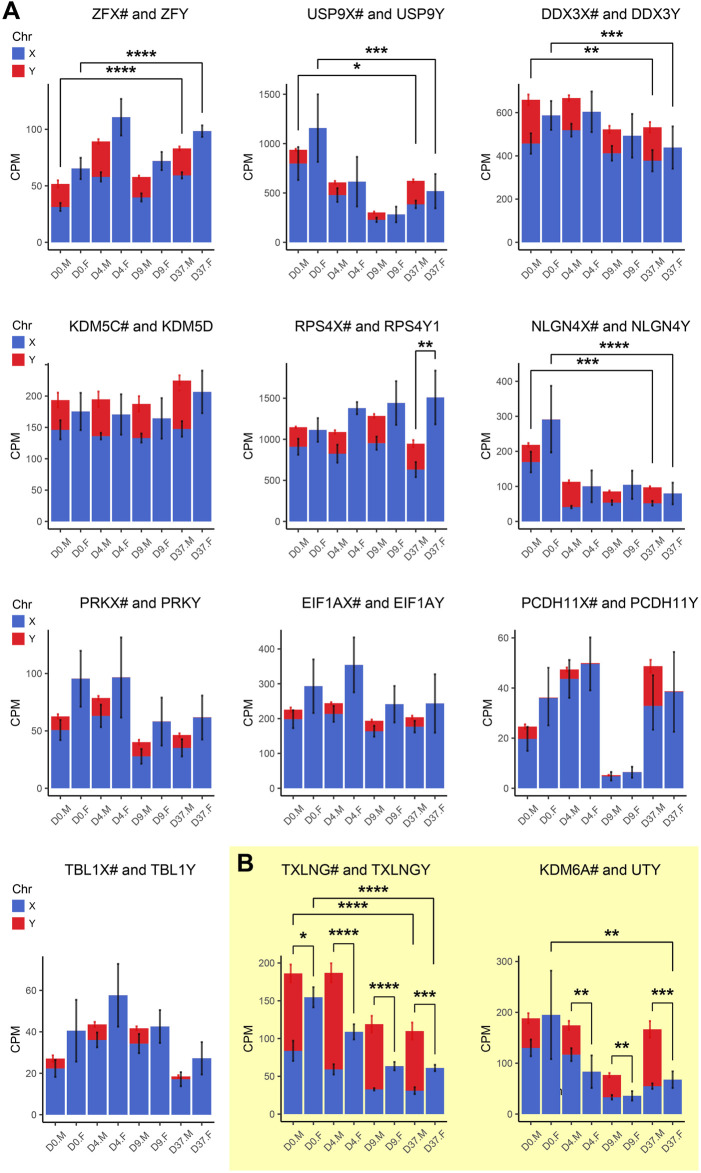
Gametologous genes with high Y homolog expression: Gene expression was analyzed based on total RNA sequencing data. The hashtag symbol after the X homolog indicates that the gene is escaping XCI. Panel **(A)** shows gene expression of 10 gametolog pairs that show substantial Y chromosome homolog contribution to gene dosage balance (ZFX/Y, USP9X/Y, DDX3X/Y, KDM5C/D, RPS4X/Y1, NLGN4X/Y, PRKX/Y, EIF1AX/Y, PDCDH11X/Y, TLX1X/Y). A sex difference was noticed in RPS4X/Y, with an increased gene dosage in female cell lines (*p* < 0.01). Panel **(B)** displays two gene pairs (TXLNG/Y, KDM6A/UTY) that display excessive Y chromosome homolog expression leading to an unbalanced gene dosage. For TXLNG/Y, this led to a male bias in gene dosage at all time points D0, D4, D9, and D37 (*p* < 0.05, *p* < 0.0001, *p* < 0.0001, *p* < 0.001, respectively). For the gene pair KDM6A/UTY, a male-biased gene dosage was found in D4, D9, and D37 (*p* < 0.01, *p* < 0.01, *p* < 0.001, respectively). Differences between the male and female samples were measured using repeated measurements two-way ANOVA with Šidák’s multiple comparisons test.

Gametologous genes are X/Y homologous genes located on opposite sex chromosomes in non-recombining regions. Gene dosage of gametologous genes can contribute to sex-biased gene expression. The analysis of these genes in male and female cell lines has revealed several sex differences (Table 4). Since the impact of XCI erosion on the gene expression of X-linked genes cannot be entirely ruled out, the interpretation of gametologous gene expression should be done with caution, particularly when there is a significantly increase of expression in the X homologs in this section. We categorized the gametologous gene pairs into four groups based on their expression pattern during the neural differentiation period from D0 to D37. Group A includes genes that are significantly downregulated at D37 (*GYG2/P1*, *ARSL/P1*, *ANOS1/P2*, *GPR143/P*, *RBMX/Y1A1*; Figure 7A). In contrast, Group B comprises genes upregulated at D37 (*ARDS/P1*, *MXRA5/Y*, *SHROOM2/P1*, *TSPYL2/TSPY*, *CASK/P1*; Figure 7B), while Group C shows no change in gene expression from D0 to D37 (*STS/P1*, *TMSB4X/Y*, *BCOR/1*, *SOX3/SRY*, *OFD1X/P1Y*; Supplementary Figure S6). Group D includes all gene pairs that show substantial Y chromosome homolog expression contributing to gene dosage compensation (*ZFX/Y*, *USP9X/Y*, *DDX3X/Y*, *KDM5C/D*, *RPS4X/Y1*, *NLGN4X/Y*, *PRKX/Y*, *EIF1AX/Y*, *PCDH11X/Y*, *TLX1X/Y*; Figure 8A). This group includes two gene pairs (*TXLNG/Y*, *KDM6A/UTY)* that display a significant overexpression of the Y chromosome homolog leading to sex biases in gene dosage, Figure 8B. More than half of the X-linked genes of the X/Y homologous gene pairs (19 out of 27) are escaping XCI according to Katsir and Linial (2019), Garieri et al. (2018) and Zhang et al. (2013). We observe that for many of the XCI-escapees (12 out of 19), the Y chromosome compensates the increased X chromosome expression, and therefore, these gene pairs do not contribute to sex differences. These genes were clustered in Group D, with the exception of one gene (*ANOS1*), which sharply drops in gene expression at D37 and thus is categorized into Group A.

**TABLE 4:** Grouping of X/Y homologs according their expression during neuronal differentiation and sex-bias in gene dosage.

Group A - downregulated at D37		Group C - No change in gene expression during differentiation		Group D - Y-linked gene expression leading to gene dosage compensation	
Gene	Sex-bias	Gene	Sex-bias	Gene	Sex-bias
**#**GYG2/P1	XX: D0, D4, D9	**#**STS/P1		**#**ZFX/Y	
ARSL/P1		**#**TMSB4X/Y		**#**USP9X/Y	
**#**ANOS1/P2	XX: D0, D4	BCOR/1	XX: D0, D4	**#**DDX3X/Y	
GPR143/P		SOX3/SRY	XX: D37	**#**KDM5C/D	
RBMX/Y1A1		**#**OFD1X/P1Y		**#**RPS4X/Y1	XX: D37
Group B - Upregulated at D37				**#**NLGN4X/Y	
Gene	Sex-bias			**#**PRKX/Y	
**#**ARSD/P1	XX: D0, D4, D37			**#**EIF1AX/Y	
**#**MXRA5/Y	XX: D37			**#**PCDH11X/Y	
SHROOM2/P1				**#**TBL1X/Y	
TSPYL2/Y1				**#**TXLNG/Y	XY: D0, D4, D9, D37
CASK/P1				**#**KDM6A/UTY	XY: D4, D9, D37

**#** marks genes escaping XCI, XX (red) and XY (blue) displays increased gene dosage in female or male hESC, lines, respectively.

We detected a female-biased overexpression of gametologous genes in Group A (*GYG2*, *ANOS1*, *BCOR1*) during early differentiation (D0, D4, D9). This sex bias could contribute to different prerequisites that affect subsequent differentiation steps. We also noticed a female-biased overexpression in genes of Group B: *ARSD* at D0, D4, and D37 and *MXRA5* at D37. A sex bias in Group B at D37 suggests relevance in late neural differentiation or maintenance. Of the Group C genes, the gene *RPS4X* is significantly overexpressed in females at D37 due to insufficient Y chromosome gene dosage compensation. Moreover, the Y homolog expression of the Group D gene pairs *TXLNG*/*TXLNGY* and *KDM6A*/*UTY* is very high at nearly all timepoints, leading to a significant increased gene dosage in male cell lines.

## Discussion

Understanding sex differences in the field of neuroscience is important to predict, monitor and treat neurological disorders, especially since a majority of early-onset neurodevelopmental disorders show a clear sex bias (Boyle et al., 2011; Loke et al., 2015; Tesic et al., 2019). In this study, we sought to determine sex-specific gene expression during early neural differentiation. We used four male and four female-derived hESC lines and applied a differentiation protocol resulting in a population of mixed neurons. Using hESCs as a research model has several advantages. By using epiblast-derived embryonic stem cells, we aimed to keep the epigenetic variability low, that can otherwise arise as a result of “the epigenetic memory” of the cell-of-origin when cells are reprogrammed to an induced pluripotent state (Narsinh et al., 2011). Furthermore, we can exclude the effects of sex hormones by using a cell culture model in a controlled *in vitro* environment. Thus, we consider our model a valuable system to study genetic sex differences that affect early nervous system development. It proved to be a robust system as it resulted in reproducible and comparable differentiation in all cell lines (Figures 1B–E). While our focus was primarily on neurons, it is important to acknowledge the complexity of neural development, which encompasses various cell types such as astrocytes, oligodendrocytes and microglia. Our findings demonstrate that under the culture conditions employed, neuronal development was favoured, with no discernible markers for other cell types within the neuronal lineage detected. While it's important to interpret these findings thoughtfully, it's worth noting that the culture conditions utilized, though not exact replicas of organotypic environments, still provide valuable insights contributing significantly to our understanding of sex-specific mechanisms in neuronal differentiation.

While X-linked genes have already been extensively linked to the development and functions of the nervous system (Skuse, 2005; Nguyen and Disteche, 2006; Mallard et al., 2021; Raznahan and Disteche, 2021; Cabrera Zapata et al., 2022), evidence for the involvement of Y-linked genes in male neuronal development is scarce (Vakilian et al., 2015; Meyfour et al., 2017; Johansson et al., 2019; Pottmeier et al., 2020; Cabrera Zapata et al., 2022). In this study, we confirm the involvement of X- and Y-linked genes in the neuronal differentiation of male and female human embryonic stem cells. Furthermore, we provide evidence for sex-biased gene expression during neural differentiation, most prominent after 37 days of differentiation, and highlight 13 candidate genes that show sex-biased expression, thus possibly impacting neuronal development. Finally, we display gene dosage compensation of X homologs that escape X-inactivation through their Y homolog in male cell lines suggesting a more important role for the Y chromosome in neurodevelopment than generally believed.

The comparison of gene expression in male and female stem cell lines presents a number of challenges. One of them, which specifically affects X-linked gene expression is the state of XCI in female stem cell lines. It is well-documented that commonly used cell culture conditions lead to the erosion of XCI in female cell lines (Hanna et al., 2010; Cloutier et al., 2022), which in turn can result in increased expression of X-linked and autosomal genes. The absence of XIST expression in two of the female cell lines and the number of genes with an XX:XY expression ratio above 1.1 indicates different XCI erosion statuses in our cell lines. However, despite this erosion, the very similar expression of X-linked genes and the low number of X chromosome genes with an XX:XY expression ratio above 1.1 indicates that the XCI erosion has only a minor effect on their X-linked gene expression. The same is true for the autosomal gene expression as the comparison of the average X:A ratio revealed no significant increase in the XIST positive cell line. In addition, our female cell lines were able to differentiate equally well to neurons, whereas under XCI erosion cell lines have been reported to be impaired in neural differentiation (Motosugi et al., 2022). Moreover, we must consider whether our analyses to detect sex differences in the study might be influenced by genes exhibiting high variance, possibly attributed to the erosion of XCI. Here, it's worth noting that such genes were excluded based on the stringent filtering criteria we implemented for identifying sex-biased genes. Consequently, we assert that the findings of our study remain unaffected by the varying XCI erosion statuses observed in the XX cell lines.

The use of the XX:XY ratio to estimate the number of genes affected by XCI erosion is an approximation and may not cover all cases. The observed disparities for chromosomes X, 5, and 16 reflect a combination of cell-line-specific variability and authentic effects of XCI erosion, which are inherently intertwined and indistinguishable. Certain scenarios may remain unaccounted for, such as instances where physiologically low X-linked gene expression in a female cell line, yielding an XX:XY ratio of 0.5, is subsequently doubled due to the reactivation of the second X chromosome through XCI erosion. In this scenario, although the gene is affected, it does not meet the criteria for being considered “affected by XCI erosion” (ratio>1.1). This methodology assumes uniform X-linked gene expression between female and male cells under identical experimental conditions. We consider this approach suitable to estimate the general progression of XCI erosion and its impact on X-linked gene expression in female cell lines, given that average X-linked expression levels are commonly reported to be elevated in XIST^–^hPSCs compared to XIST^+^ and male hPSCs (Bar et al., 2019; Motosugi et al., 2022; Sarel-Gallily and Benvenisty, 2022). Nevertheless, results including X-linked genes should be interpreted with caution as a possible effect from XCI erosion cannot be entirely excluded.

Considering that the majority of gametologous genes are XCI escape genes (19 out of 27), transcribed from two X chromosomes, it is improbable that XCI erosion significantly affects their gene expression. Only two gametologous genes (*CASK* and *SHROOM2*) exhibit signs of XCI erosion, as indicated by an XX:XY ratio above 1.1 and are not XCI escape genes. Therefore, we consider the analysis of gametologous genes presented in this study to be largely unaffected by XCI erosion.

### Genetic sex differences are not routinely investigated

Even though ‘sex differences’ is a subject that receives increasing awareness among researchers and is regularly debated among neuroscientists (DeCasien et al., 2022), the investigation of sex-biased gene expression is not pursued frequently. Most studies that investigate sex-biased gene expression during neurodevelopment are transcriptomic studies that utilize brain tissues of the developing embryo (Shi et al., 2016; O’Brien et al., 2018; Johansson et al., 2019; Liu et al., 2020; Oliva et al., 2020; de Toledo et al., 2022). We have only found one recent study that, like ours, uses human stem cell lines to investigate gene expression during neural differentiation (Waldhorn et al., 2022). Unfortunately, they only used 1 cell line per sex. Many studies that use multiple human stem cells for neuronal differentiation investigate differentiation characteristics and mechanisms but do not include or consider the sex of the used cell lines (Maroof et al., 2013; Tsankov et al., 2015; Bardy et al., 2016; Hoffman et al., 2017; Schwartzentruber et al., 2018; Burke et al., 2020; Kathuria et al., 2020; Lindhout et al., 2020; Strano et al., 2020; Kuruş et al., 2021). Even large-scale cell bank initiatives that meticulously characterize their stem cells do not investigate differences or similarities between the cell lines of different sexes (Salomonis et al., 2016; Kilpinen et al., 2017; Panopoulos et al., 2017; D’Antonio et al., 2017). This underlines that sex differences are still not routinely investigated even though the transcriptomic data is at hand. A large number of studies use only a single cell line for the investigation of neuronal differentiation properties, neglecting to account for interindividual differences of cell lines and missing the chance to investigate sex differences (Wu et al., 2010; van de Leemput et al., 2014; Chu et al., 2016; Close et al., 2017; Song et al., 2017; Meisig et al., 2020; Allison et al., 2021; Solomon et al., 2021).

### Y-linked genes in neuronal development: Insights from the differentiation of male embryonal stem cells

It has been reported that a major contribution to sex differences arises from the Y chromosome genes or the dosage of X chromosome genes (Jazin and Cahill, 2010). The Y-linked genes detected during our differentiation experiment seem to be robustly expressed since most of them were also found in undifferentiated male human iPSCs (13 out of 16, compared with D0) as well as after 14 days of neuronal differentiation (14 out of 17, compared with D9 and D37) (Waldhorn et al., 2022). Based on their gene expression patterns, a number of Y-linked genes (Figure 2A; Supplementary Table S4) are likely to be involved in the development of neurons in male stem cell lines. The peaking expression of the genes *TXLNGY*, *NLGN4Y* and *ZFY* at D4 indicates a role in early neuronal induction. Interestingly, *TXLNGY* is considered a pseudogene but shows expression in multiple tissues, including the brain. To date, the function of *TXLNGY* is still unknown. Expression evidence from our present and a previous study (Pottmeier et al., 2020) both suggest an involvement of *TXLNGY* in processes of early neuronal induction. The gene *NLGN4Y* is a member of the neuroligin family, which is important for the formation and function of synapses. Due to the high sequence similarity of *NLGN4X* and *Y* in humans (97%), the two homologs are assumed to have the same function. A recent study, however, showed that a single amino acid difference in *NLNG4Y* results in a deficit in trafficking that leads to decreased surface expression and thus possibly to decreased synaptic function of NLGN4Y (Nguyen et al., 2020). Nevertheless, *NLGN4Y* has been reliably detected in the human brain (Trabzuni et al., 2013; Tukiainen et al., 2017; Johansson et al., 2019) and during neuronal differentiation of stem cells (Pottmeier et al., 2020). Our results support the claims for an involvement of *NLGN4Y* in neuronal development and suggest a role in early neuronal induction. The gene *ZFY* encodes a zinc finger transcription factor that is able to regulate gene expression and is expressed in the developing and mature human brain (Mayer et al., 1998; Weickert et al., 2009; Shi et al., 2016). The role of *ZFY* in neuronal differentiation is unknown. In our experiment, *ZFY* is peaking at D4 of neuronal differentiation, indicating a possible contribution to the early neuronal induction of male stem cells. The gene *RPS4Y1* is the only Y-linked gene peaking in expression at D9. RPS4Y1 is a ribosomal protein and thus involved in the synthesis of proteins. Its function in neuronal development is not fully understood; however, it is expressed in the developing and mature brain (Weickert et al., 2009; Kang et al., 2011; Shi et al., 2016; Khani et al., 2022). Moreover, it is reported to play a role in neuronal differentiation (Vakilian et al., 2015), which is supported by our results, specifically in an advanced stage of neuronal induction. The Y-linked genes with the highest expression at D37 are *USP9Y*, *UTY*, *KDM5D* and *PCDH11Y*. Due to their peaking expression at this timepoint, these genes are likely to play an important role at this stage of differentiation. The gene *USP9Y* encodes a deubiquitinase and is expressed in multiple tissues, including the developing and mature brain (Weickert et al., 2009; Kang et al., 2011; Shi et al., 2016; Johansson et al., 2019). Ubiquitylation orchestrates core signaling networks essential for stem cell maintenance and differentiation. Consistent with our findings, there is evidence suggesting that USP9Y plays a role in neuronal differentiation (Vakilian et al., 2015). *PCDH11Y* encodes a member of the protocadherin protein family. Due to their high sequence similarity (98%), *PCDH11X* and its Y chromosome homolog are believed to perform a similar function. Many protocadherins are predominantly expressed in the brain and play a role in cell-cell adhesion and signaling, specifically in developing neurons (Pancho et al., 2020). *PCDH11Y* is expressed in developing and adult brain tissue (Mayer et al., 1998; Weickert et al., 2009; Shi et al., 2016) and is thought to play a role in neuronal development and function through its contribution to the formation and maintenance of synapses (Durand et al., 2006). The large increase of *USP9Y* and *PCDH11Y* expression at D37 of the differentiation experiment suggests a role in neuronal maturation or maintenance.

### Histone demethylases of the Y chromosome in neurodevelopment

Histone modifications play critical roles in a plethora of biological processes, including neuronal development. We found that two Y-linked histone demethylases, UTY (KDM6C), and KDM5D (JARID1D), are highly expressed in male human embryonic stem cells after 37 days of neuronal differentiation (Figure 2A), indicating a contribution to neuronal development. Histone demethylases are particularly interesting for gene expression because they can regulate transcription initiation, elongation, and termination through post-translational histone modification. The gene *UTY (KDM6C)* and its X chromosome homolog *UTX (KDM6A)* are members of the histone lysine demethylase subfamily 6 (KDM6). It has not been fully clarified if UTY possesses catalytical activity. There are studies confirming (Walport et al., 2014) but also denying (Hong et al., 2007; Lan et al., 2007; Shpargel et al., 2012) UTY its catalytical ability. UTY seems to have reduced catalytic activity compared to UTX, but has the same protein binding capacity (Walport et al., 2014). It is possible that UTY has access to additional cofactors *in vivo* that support its histone demethylase activity, explaining its reduced enzymatic activity *in vitro*. Apart from their demethylating function, it has also been suggested that UTY and UTX possess a histone demethylase-independent function, as the presence of UTY allows UTX-deficient mouse embryos to survive until birth (Shpargel et al., 2012). *UTY* and *UTX* share 88% sequence identity, and both are H3K27-specific demethylases that can supports the activation of gene transcription (Hong et al., 2007; Lee et al., 2007; Swigut and Wysocka, 2007). It has been demonstrated that the regulation of H3K27 methylation states is essential for the development and function of the mammalian nervous system and that enzymes involved, such as UTX and likely also UTY, are key regulators of human neural differentiation in stem cells (Lei and Jiao, 2018; Yang et al., 2019; Shan et al., 2020; Tang et al., 2020). Our study supports the previous claims suggesting a contribution of UTY to neural differentiation.

The gene *KDM5D (JARID1D* or *SMCY)* and its X chromosome homolog *KDM5C (JARID1C* or *SMCX)* are members of the histone lysine demethylase subfamily 5 (KDM5). *KDM5D* and *KDM5C* show an 85% sequence similarity. They exhibit conserved protein domains for histone demethylase activity (JmjC), but experimentally determined H3K4 demethylase was lower for KDM5D than KDM5C (Iwase et al., 2007). A demethylation at H3K4 is considered as a sign of repressed gene transcription (Barski et al., 2007). H3K4 methylation states are regulated by several SET-domain-containing enzymes, including the KDM5/JARID1 family of histone demethylases (Cloos et al., 2008). A dysregulation of H3K4-specific methylation states has been linked to various developmental disorders and impaired cognition (Bale et al., 2010; Millan, 2013; Ronan et al., 2013; Kim et al., 2017). There is evidence that H3K4 methylation landscapes in the human cerebral cortex are dynamically regulated during prenatal development and throughout early childhood years until adolescence (Shulha et al., 2013). Furthermore, it has been shown that neuron-specific H3K4me3 peaks exist in humans, and that they are enriched at promoters of genes that control synaptic function (Cheung et al., 2010). Mutations in the X chromosome demethylase KDM5C are associated with X-linked intellectual disability and epilepsy (Strømme et al., 2002; Najmabadi et al., 2011; Poeta et al., 2013). Further, KDM5C is implicated in the regulation of neural fate commitment in stem cells (Dey et al., 2008; Kim et al., 2018). Y-linked demethylases are among the least studied enzymes involved in histone modifications, most likely because they are thought to have a lower catalytic activity than their X-linked counterparts. In our present study, we show that the Y-linked demethylases *KDM5D* and *UTY* are increasingly expressed during neuronal differentiation of human embryonic stem cells. These results are in line with a previous study, in which we have observed an increased expression of *KDM5D* and *UTY* during differentiation of neural stem cells (Pottmeier et al., 2020). Our studies suggest a role of Y-linked demethylases in male-specific neural development. Due to their conserved protein domains, it is likely that the X and Y chromosome homologous demethylases perform a similarly important function in human neural development.

### Hormone-independent genome-wide gene expression changes during neuronal differentiation

At all timepoints investigated, including the ESC stage at D0, we found the number of sex-biased genes to be increasing as differentiation progressed (Table 2). This indicates that sex differences in gene expression become more pronounced as the nervous system develops. Together with the increasing influence of sex hormones during development, this could be a major factor contributing to the emergence of sex-specific neural circuits in human brain development. Although the number of differentially expressed genes at D0 was small (65), robust sex differences were already present, and interestingly, they originated to a large part from the Y chromosome in male ESCs (16 DEG) and from the X chromosome in female ESCs (21 DEG). A gene set enrichment analysis found that male cells at this early stage are enriched for ribosomal and mitochondrial functions (Supplementary Figure S5). This could point towards increased cell proliferation and differentiation in the male cell lines. Although we have not measured the growth rate in the used hESC lines, this is in line with previous observations where we have seen increased cell proliferation in male neural stem cells (Pottmeier et al., 2020). While the number of sex chromosome genes with sex-biased expression remained constant during differentiation, the number of autosomal genes with sex-biased expression increased in both sexes during differentiation, but to a greater extent and with larger variations in male cells (Table 2).

A sex-bias in autosomal genes has been reported in a number of transcriptomic studies of neurodevelopment (Trabzuni et al., 2013; Ronen and Benvenisty, 2014; de Toledo et al., 2022). In male cells at D37, the number of autosomal genes contributes the most to the sex bias in expression (86%, 89 of 104 genes), while in the female cells, a similar number of autosomal (23) and X-linked genes (47%, 21 of 44) are displaying a sex bias in expression. It has been demonstrated that the sex chromosome complements and especially the Y-linked gene *SRY* has an effect on global gene expression. The effect is believed to be achieved by the direct activation of key autosomal genes and the autocrine or paracrine effects of sex steroids (Wijchers et al., 2010; Ronen and Benvenisty, 2014). Based on this, it is likely that a gene expression amplifying effect by the Y chromosome is also present during differentiation and could therefore explain the high number of sex-biased autosomal genes during differentiation in our male cell lines. From D0 to D4, the number of upregulated autosomal genes increased by the factor 10 in males and by the factor 3 in females, affecting biological processes related to actin dynamics in male cells and cilia, chromatin and DNA packaging in female cells, according to GSEA (Figure 4). This displays that sex differences in gene expression are already present as early as 4 days after differentiation, and that they are able to affect biological processes relevant for neuronal development (Figure 4). At D9, no gene sets were enriched in male or female cell; however, females displayed 5 times more autosomal DEG than males. At D37, the most advanced stage in our neurodevelopment experiment, we see a total of 148 sex-biased DEG from which a majority (70%) was upregulated in males. Of these DEG, 39 were implicated in neuronal processes, with the majority involved in neurodevelopment. In total, four times more DEG were upregulated in male than in female cell lines. These results are supported by Waldhorn et al. (2022) who also found the majority of DEG to arise from male cells in neuronal differentiation of hiPSC, as well as by Shi et al. (2016) providing evidence for a male-driven sex bias in the transcriptome of the human fetal brain. Our data illustrates how early sex chromosome activity can set the stage for a later manifestation of differences and suggests a larger effect on the male cell lines. While in our study the male DEG at D37 were specifically associated with neurogenesis, cell-cell signaling, synaptic transmission, trans-synaptic signaling and neuronal differentiation, transcriptome investigations of human embryos revealed contrasting results. In two other studies, female samples were enriched for neuronal differentiation and synapse specification, as well as synaptic processes and synaptic signaling, respectively (O’Brien et al., 2018; de Toledo et al., 2022). These discrepancies can be explained by the nature of the samples and the difference in stages of neuronal development, as data from the above-mentioned studies is gathered from mid-gestation embryos, that represent a more advanced stage than our hESC cells after 37 days of differentiation. A limitation of our study is the lack of RNA sequencing data from additional timepoints during differentiation. Even though data was collected over a number of timepoints until the endpoint at D37, for the interpretation of results from the latest stage it has to be considered that possible delays or shifts in timing in neuron development between the sexes cannot be resolved. Moreover, the results are retrieved from pooled material from a mixture of cells and thereby limiting conclusions on the single-cell or cell-type basis.

Among the sex-biased DEG, we found a considerable number of X-linked genes throughout all differentiation stages (Supplementary Table S1). Most notably are the 12 genes *ARSD*, *CTPS2*, *GYG2*, *LRCH2*, *NAP1L3*, *RBBP7*, *SYAP1*, *TXLNG*, *ZFX*, *ZRSR2*, *PLS3,* and *PABPC5* which show a significant overexpression in female cell lines in every investigated timepoint (D0, D4, D9, D37). All of these genes, except *PLS3*, *NAPL1L3*, *PABPC5,* and *LRCH2,* have been shown to stably escape X inactivation (Katsir and Linial, 2019), explaining the consistent sex bias in expression during differentiation. *PLS3*, *NAP1L3,* and *PABPC5* have been described as variable XCI, dependent on the investigated tissue type (Garieri et al., 2018). The genes *TXLNG* and *ZFX* are gametologous genes that are significantly overexpressed genes in females. However, we have shown that their Y-linked homolog compensates for the overexpression (Figure 8) leading to relatively balanced gene dosage. Of the above-mentioned X-linked genes with a significant overexpression in females *PLS3*, *SYAP1*, *TXLNG*, *ZFX* show an implication in neurodevelopment according to GO-terms and the genes *ARDS*, *LRCH2,* and *ZRSR2* are peaking in expression at D37, also suggesting an involvement in neuronal differentiation. Interestingly, the X-linked genes *GABRE* and AC244197.3 show a significant overexpression in male cell lines at D37. AC244197.3 encodes a protein of unknown function. Since AC244197.3 significantly increases in gene expression at D37, it suggests an involvement in male-specific neuronal differentiation or maintenance. *GABRE* encodes the epsilon subunit of the gamma-aminobutyric acid A receptor. During neurodevelopment, GABA plays a critical role in regulating the proliferation, differentiation, and migration of neural cells. GABA also plays a role in the formation and maturation of synapses. GABA_A_ receptor is constructed as a pentameric structure of multiple subunits (α, β, γ, δ, ɛ, θ, π). Sex differences in GABA receptor subtypes were already reported in adult human brain tissue, displaying a higher level of GABA_A_ receptor subunits α1, α2, α5, and β3 in male brain tissue (Pandya et al., 2019).

### Compensation of sex differences by gametologous genes

Closer investigation of our RNAseq data for gene dosage in gametologous genes (Figures 7, 8; Supplementary Figure S6) revealed that seven gene pairs showed a higher gene dosage in female cells, with GYG2/P1 and ARSD/P1 higher expressed over several stages, ANOS1/P2 and BCOR/1 at early, and MXRA5/Y, RPS4X/Y1, and SOX3/SRY at the last stage, D37. Two gametologous pairs, TXLNG/Y and KDM6A/UTY showed a higher gene dosage in male cells at all differentiation timepoints due to the contribution of the Y homolog. *TXLNG/Y* was also overexpressed in undifferentiated ESCs (D0). In a previous study, we found *UTY* and also *KDM5D* expression increased upon differentiation in male NSCs (Pottmeier et al., 2020). Interestingly, the gene dosage of the X homolog *KDM5C* is compensated by the Y homolog even in the undifferentiated ESC stage. The lysine demethylases KDM6A target the Hox gene family and thereby control pluripotency and lineage-specific genes and can be partly compensated by the complement UTY. Despite or rather because of the critical function in development, *KDM6A*/*UTY* might be an important contributor to the genetically driven sexual dimorphisms.

### Candidate sex-biased genes implicated in neurodevelopment

We annotated the sex-biased DEG at D37 with abundant expression using the following data: GO-terms related to neurodevelopment, upregulation at 37 days of differentiation, enrichment in clusters of neuronal processes in GSEA, a minimum expression level of 500 DESeq2 normalized counts, and presence in literature associated with neurodevelopmental processes (Supplementary Table S5). This has resulted in 10 candidate genes that show upregulated expression in male cell lines (*NHLH2*, *EBF1*, *SLC17A6*, *RUNX1T1*, *KIF5A*, *AKAP12*, *MDGA1*, *ONECUT2*, *P2RX3*, *LMX1B*) and 3 candidate genes with upregulated expression in female cell lines (*SYAP1*, *AMOT*, *PAK3*), Table 3. The observed sex differences in candidate genes have proved to be highly replicable even across gene expression analysis methods, demonstrated by the consistent results of RNA sequencing (Supplementary Figure S7) and qPCR analysis of replicated differentiation experiments (Figure 6). Noticeably, all of the genes with higher expression in male cell lines are autosomal genes and half of them are brain-specific transcription factors (*NHLH2*, *EBF1*, *RUNX1T1*, *ONECUT2*, *LMX1B)* with critical functions in the development and differentiation of neurons. In addition, all of the candidate genes in males are implicated in neurodevelopmental and/or neurological diseases (Huynh et al., 2012; Villaescusa et al., 2016; Afshar et al., 2020; Sherman et al., 2022; Flex et al., 2023; Kimura et al., 2023; Verma et al., 2023). The three candidate genes that are upregulated in female cells are X-linked genes, highlighting the importance of the X-chromosome in neurodevelopment. All of these genes are known to be involved in neurological diseases; *AMOT* is implicated in Alzheimer’s disease and *PAK3* is associated with inherited X-linked intellectual disability (Sherman et al., 2022). The human gene encoding SYAP1 is located within chromosomal band Xp22.2, a region associated with mental retardation, developmental delay, and autism spectrum disorder (Sismani et al., 2011; Prasad et al., 2012). The candidate genes display varying degrees of conservation; all are conserved among mammals, with confirmed expression and function in the mouse nervous system. Additionally, some genes show high conservation with orthologues not only in mice but also in other model organisms. For instance, *LMX1B*, *KIF5A*, and *P2RX3* have orthologues in zebrafish, while *ONECUT2*, *RUNX1T*, *KIF5A*, *SLC17A6*, *SYAP1*, and *PAK3* have orthologues in *drosophila*. Moreover, *LMX1B*, *SLC17A6*, and *PAK3* have orthologues in *C. elegans*. All these genes exhibit confirmed expression and a role in the development and/or function of the nervous system across multiple species (Wigerius et al., 2020; Bult and Sternberg, 2023), likely suggesting that they have roles fundamental in pathways conserved across a wide range of organisms.

Strikingly, not only the number of DEG in male cells is higher, there are also a considerable number of transcription factors among the candidate genes. In contrast, the candidate genes in female cells are associated with cellular processes and signaling while transcription factors are not overrepresented. This could point towards an active process in male cells based on intrinsic genetic factors that contribute to early programming or priming of the neural cells. Notably, as mentioned earlier, the direct influence of *SRY* on global gene expression emerges as a significant genetic contributor to sex-specific expression patterns. Epigenetic modifiers, such as histone demethylases like *KDM6A*, *KDM5D*, and *UTY*, have been identified throughout the differentiation phase, with *UTY* exhibiting markedly elevated levels in male cells compared to female cells. This discrepancy potentially impacts the chromatin landscape surrounding the promoters and enhancers of candidate genes. Using databases at The Biological General Repository for Interaction Datasets (BioGRID4.4 https://thebiogrid.org/) we uncovered several documented direct or mediated protein-protein interactions between epigenetic modifiers and DEG: *UTY* displayed interactions with *KDM6B*, *ANOS*, *KIF*-paralogues, and *USP9X*, while *KDM5D* exhibited interactions with *AMOT*, *Runx1*, *KDM6A*, and *NHLH*-paralogues. *TXLNGY* was found to interact with *AMOT* and *KIF*-paralogues via *TRIM14*. Additionally, evidence of interactions between *KDM6A* and *AMOT*, *NHLH*- and *KIF*-paralogues, *USP9*, and *EIF1AY* was observed. Genetically, interactions were documented between KDM6A and KDM5A and 5C. Moreover, *KDM6A* was observed to directly interact with the pioneer transcription factor FOXA1, as well as with PAX, LHX, GATA, and SOX family factors. These findings suggest a complex involvement of epigenetic regulatory networks, intertwined with fundamental neurodevelopmental pathways, influencing the expression of sex-biased genes. However, it remains to be determined whether *UTY*, sharing 82% sequence homology with its gametologue *KDM6A*, shares the same interaction partners. The significant difference in the number of known interactors between X-gametologues, particularly KDM6A (with 158 interactors), and the Y-gametologue *UTY* (with 21 interactors), warrants further investigation into the underlying mechanisms governing sex-specific gene expression. The fewer interactors found for Y-linked gene products may stem from a lack of comprehensive information, potentially due to the overlooked roles of Y-chromosome genes.

These sex differences in early programming could set the stage for later differentiation events and contribute to the establishment of a neural network distinct from that in females. Additionally, the genetic differences observed could prepare for later responsiveness to hormonal cues during further development or in response to external stimuli. The observed expression bias may contribute not only to physiological sex-specific differences but also to varying susceptibility to neurological diseases as deviations in proliferation and differentiation have been associated with neurodevelopmental disorders (Ernst, 2016) that have a higher prevalence in males such as autism spectrum disorder, schizophrenia, attention-deficit hyperactivity disorder have a higher prevalence in males (Saha et al., 2005; May et al., 2019; Maenner et al., 2020).

Further research is needed to elucidate the specific molecular mechanisms underlying these observations and to understand how they contribute to the development of sexually dimorphic characteristics in the nervous system.

## Conclusion

In conclusion, our study identified a very early genetically driven and hormone-independent trait separation according to sex in the neurodifferentiation of human embryonic stem cells. We provide a catalog of genes that display a large degree of sex differences in expression during neuronal differentiation, which holds significant implications for understanding the origins of sex-biased neurodevelopmental diseases. The most substantial contribution was found to arise from the male transcriptome, involving both Y chromosome and autosomal genes, of which the majority have crucial functions in neurodevelopment and differentiation. It is important to note that mRNA and protein levels do not always correlate, and additional layers of post-transcriptional regulation are likely influenced by sex as well. Therefore, we advocate for further investigation into the direct impact of sex on protein expression. To fully validate these findings, additional research is warranted to confirm the observed sex biases and explore the potential functional roles of candidate genes in neuronal differentiation. Ultimately, we hope that our study contributes to the understanding of genetic sex differences in neurodevelopment and sheds light on their potential implications in the development of sex-biased neurological disorders.

## Data Availability

The data presented in the study are deposited in the Gene Expression Omnibus (GEO) repository, accession number GSE249035.

## References

[B1] Afshar H. Adelirad F. Kowsari A. Kalhor N. Delbari A. Najafipour R. (2020). Natural selection at the NHLH2 core promoter exceptionally long CA-repeat in human and disease-only genotypes in late-onset neurocognitive disorder. Gerontology 66 (5), 514–522. 10.1159/000509471 32877896

[B2] Allison T. Langerman J. Sabri S. Otero-Garcia M. Lund A. Huang J. (2021). Defining the nature of human pluripotent stem cell-derived interneurons via single-cell analysis. Stem Cell Rep. 16 (10), 2548–2564. 10.1016/j.stemcr.2021.08.006 PMC8514853 34506726

[B3] Arnold A. P. (2017). A general theory of sexual differentiation. J. Neurosci. Res. 95 (1-2), 291–300. 10.1002/jnr.23884 27870435 PMC5369239

[B4] Arnold A. P. McCarthy M. M. (2016). “Sexual differentiation of the brain and behavior: a primer,” in Neuroscience in the 21st century. Editors Pfaff D. W. Volkow N. D. (New York, NY: Springer New York), 2139–2168.

[B5] Bale T. L. Baram T. Z. Brown A. S. Goldstein J. M. Insel T. R. McCarthy M. M. (2010). Early life programming and neurodevelopmental disorders. Biol. Psychiatry 68 (4), 314–319. 10.1016/j.biopsych.2010.05.028 20674602 PMC3168778

[B6] Bar S. Seaton L. R. Weissbein U. Eldar-Geva T. Benvenisty N. (2019). Global characterization of X chromosome inactivation in human pluripotent stem cells. Cell Rep. 27 (1), 20–29.e3. 10.1016/j.celrep.2019.03.019 30943402

[B7] Bardy C. van den Hurk M. Kakaradov B. Erwin J. A. Jaeger B. N. Hernandez R. V. (2016). Predicting the functional states of human iPSC-derived neurons with single-cell RNA-seq and electrophysiology. Mol. Psychiatry 21 (11), 1573–1588. 10.1038/mp.2016.158 27698428 PMC5071135

[B8] Barski A. Cuddapah S. Cui K. Roh T. Y. Schones D. E. Wang Z. (2007). High-resolution profiling of histone methylations in the human genome. Cell 129 (4), 823–837. 10.1016/j.cell.2007.05.009 17512414

[B9] Boyle C. A. Boulet S. Schieve L. A. Cohen R. A. Blumberg S. J. Yeargin-Allsopp M. (2011). Trends in the prevalence of developmental disabilities in US children, 1997-2008. Pediatrics 127 (6), 1034–1042. 10.1542/peds.2010-2989 21606152

[B10] Bult C. J. Sternberg P. W. (2023). The alliance of genome resources: transforming comparative genomics. Mamm. Genome 34 (4), 531–544. 10.1007/s00335-023-10015-2 37666946 PMC10628019

[B11] Burke E. E. Chenoweth J. G. Shin J. H. Collado-Torres L. Kim S. K. Micali N. (2020). Dissecting transcriptomic signatures of neuronal differentiation and maturation using iPSCs. Nat. Commun. 11 (1), 462. 10.1038/s41467-019-14266-z 31974374 PMC6978526

[B12] Cabrera Zapata L. E. Garcia-Segura L. M. Cambiasso M. J. Arevalo M. A. (2022). Genetics and epigenetics of the X and Y chromosomes in the sexual differentiation of the brain. Int. J. Mol. Sci. 23 (20), 12288. 10.3390/ijms232012288 36293143 PMC9603441

[B13] Carruth L. L. Reisert I. Arnold A. P. (2002). Sex chromosome genes directly affect brain sexual differentiation. Nat. Neurosci. 5 (10), 933–934. 10.1038/nn922 12244322

[B14] Chambers S. M. Fasano C. A. Papapetrou E. P. Tomishima M. Sadelain M. Studer L. (2009). Highly efficient neural conversion of human ES and iPS cells by dual inhibition of SMAD signaling. Nat. Biotechnol. 27 (3), 275–280. 10.1038/nbt.1529 19252484 PMC2756723

[B15] Cheung I. Shulha H. P. Jiang Y. Matevossian A. Wang J. Weng Z. (2010). Developmental regulation and individual differences of neuronal H3K4me3 epigenomes in the prefrontal cortex. Proc. Natl. Acad. Sci. U. S. A. 107 (19), 8824–8829. 10.1073/pnas.1001702107 20421462 PMC2889328

[B16] Choleris E. Galea L. A. M. Sohrabji F. Frick K. M. (2018). Sex differences in the brain: implications for behavioral and biomedical research. Neurosci. Biobehav. Rev. 85, 126–145. 10.1016/j.neubiorev.2017.07.005 29287628 PMC5751942

[B17] Chu L.-F. Leng N. Zhang J. Hou Z. Mamott D. Vereide D. T. (2016). Single-cell RNA-seq reveals novel regulators of human embryonic stem cell differentiation to definitive endoderm. Genome Biol. 17 (1), 173. 10.1186/s13059-016-1033-x 27534536 PMC4989499

[B18] Cloos P. A. C. Christensen J. Agger K. Helin K. (2008). Erasing the methyl mark: histone demethylases at the center of cellular differentiation and disease. Genes & Dev. 22 (9), 1115–1140. 10.1101/gad.1652908 18451103 PMC2732404

[B19] Close J. L. Yao Z. Levi B. P. Miller J. A. Bakken T. E. Menon V. (2017). Single-cell profiling of an *in vitro* model of human interneuron development reveals temporal dynamics of cell type production and maturation. Neuron 93 (5), 1035–1048. 10.1016/j.neuron.2017.02.014 28279351 PMC5480972

[B20] Cloutier M. Kumar S. Buttigieg E. Keller L. Lee B. Williams A. (2022). Preventing erosion of X-chromosome inactivation in human embryonic stem cells. Nat. Commun. 13 (1), 2516. 10.1038/s41467-022-30259-x 35523820 PMC9076865

[B21] Corre C. Friedel M. Vousden D. A. Metcalf A. Spring S. Qiu L. R. (2016). Separate effects of sex hormones and sex chromosomes on brain structure and function revealed by high-resolution magnetic resonance imaging and spatial navigation assessment of the Four Core Genotype mouse model. Brain Struct. Funct. 221 (2), 997–1016. 10.1007/s00429-014-0952-0 25445841

[B22] D’Antonio M. Woodruff G. Nathanson J. L. D'Antonio-Chronowska A. Arias A. Matsui H. (2017). High-throughput and cost-effective characterization of induced pluripotent stem cells. Stem Cell Rep. 8 (4), 1101–1111. 10.1016/j.stemcr.2017.03.011 PMC5390243 28410643

[B23] DeCasien A. R. Guma E. Liu S. Raznahan A. (2022). Sex differences in the human brain: a roadmap for more careful analysis and interpretation of a biological reality. Biol. sex Differ. 13 (1), 43. 10.1186/s13293-022-00448-w 35883159 PMC9327177

[B24] de Toledo V. H. C. Feltrin A. S. Barbosa A. R. Tahira A. C. Brentani H. (2022). Sex differences in gene regulatory networks during mid-gestational brain development. Front. Hum. Neurosci. 16, 955607. 10.3389/fnhum.2022.955607 36061507 PMC9428411

[B25] Dey B. K. Stalker L. Schnerch A. Bhatia M. Taylor-Papidimitriou J. Wynder C. (2008). The histone demethylase KDM5b/JARID1b plays a role in cell fate decisions by blocking terminal differentiation. Mol. Cell. Biol. 28 (17), 5312–5327. 10.1128/MCB.00128-08 18591252 PMC2519745

[B26] Durand C. M. Kappeler C. Betancur C. Delorme R. Quach H. Goubran-Botros H. (2006). Expression and genetic variability of PCDH11Y, a gene specific to *Homo sapiens* and candidate for susceptibility to psychiatric disorders. Am. J. Med. Genet. 141B (1), 67–70. 10.1002/ajmg.b.30229 16331680 PMC4867006

[B27] Edgar R. Domrachev M. Lash A. E. (2002). Gene Expression Omnibus: NCBI gene expression and hybridization array data repository. Nucleic Acids Res. 30 (1), 207–210. 10.1093/nar/30.1.207 11752295 PMC99122

[B28] Ernst C. (2016). Proliferation and differentiation deficits are a major convergence point for neurodevelopmental disorders. Trends Neurosci. 39 (5), 290–299. 10.1016/j.tins.2016.03.001 27032601

[B29] Ewels P. Magnusson M. Lundin S. Käller M. (2016). MultiQC: summarize analysis results for multiple tools and samples in a single report. Bioinformatics 32 (19), 3047–3048. 10.1093/bioinformatics/btw354 27312411 PMC5039924

[B30] Flex E. Albadri S. Radio F. C. Cecchetti S. Lauri A. Priolo M. (2023). Dominantly acting KIF5B variants with pleiotropic cellular consequences cause variable clinical phenotypes. Hum. Mol. Genet. 32 (3), 473–488. 10.1093/hmg/ddac213 36018820 PMC9851748

[B31] Garieri M. Stamoulis G. Blanc X. Falconnet E. Ribaux P. Borel C. (2018). Extensive cellular heterogeneity of X inactivation revealed by single-cell allele-specific expression in human fibroblasts. Proc. Natl. Acad. Sci. U. S. A. 115 (51), 13015–13020. 10.1073/pnas.1806811115 30510006 PMC6304968

[B32] Gegenhuber B. Tollkuhn J. (2020). Signatures of sex: sex differences in gene expression in the vertebrate brain. Wiley Interdiscip. Rev. Dev. Biol. 9 (1), e348. 10.1002/wdev.348 31106965 PMC6864223

[B33] Golden L. C. Voskuhl R. (2017). The importance of studying sex differences in disease: the example of multiple sclerosis. J. Neurosci. Res. 95 (1-2), 633–643. 10.1002/jnr.23955 27870415 PMC5825192

[B34] Hanna J. Cheng A. W. Saha K. Kim J. Lengner C. J. Soldner F. (2010). Human embryonic stem cells with biological and epigenetic characteristics similar to those of mouse ESCs. Proc. Natl. Acad. Sci. U. S. A. 107 (20), 9222–9227. 10.1073/pnas.1004584107 20442331 PMC2889088

[B35] Hoffman G. E. Hartley B. J. Flaherty E. Ladran I. Gochman P. Ruderfer D. M. (2017). Transcriptional signatures of schizophrenia in hiPSC-derived NPCs and neurons are concordant with post-mortem adult brains. Nat. Commun. 8 (1), 2225. 10.1038/s41467-017-02330-5 29263384 PMC5738408

[B36] Hong S. Cho Y. W. Yu L. R. Yu H. Veenstra T. D. Ge K. (2007). Identification of JmjC domain-containing UTX and JMJD3 as histone H3 lysine 27 demethylases. Proc. Natl. Acad. Sci. U. S. A. 104 (47), 18439–18444. 10.1073/pnas.0707292104 18003914 PMC2141795

[B37] Huynh M. T. Béri-Dexheimer M. Bonnet C. Bronner M. Khan A. A. Allou L. (2012). RUNX1T1, a chromatin repression protein, is a candidate gene for autosomal dominant intellectual disability. Am. J. Med. Genet. Part A 158A (7), 1782–1784. 10.1002/ajmg.a.35386 22644616

[B38] Iwase S. Lan F. Bayliss P. de la Torre-Ubieta L. Huarte M. Qi H. H. (2007). The X-linked mental retardation gene SMCX/JARID1C defines a family of histone H3 lysine 4 demethylases. Cell 128 (6), 1077–1088. 10.1016/j.cell.2007.02.017 17320160

[B39] Jazin E. Cahill L. (2010). Sex differences in molecular neuroscience: from fruit flies to humans. Nat. Rev. Neurosci. 11 (1), 9–17. 10.1038/nrn2754 20019686

[B40] Johansson M. M. Pottmeier P. Suciu P. Ahmad T. Zaghlool A. Halvardson J. (2019). Novel Y-chromosome long non-coding RNAs expressed in human male CNS during early development. Front. Genet. 10, 891. 10.3389/fgene.2019.00891 31608120 PMC6769107

[B41] Kang H. J. Kawasawa Y. I. Cheng F. Zhu Y. Xu X. Li M. (2011). Spatio-temporal transcriptome of the human brain. Nature 478 (7370), 483–489. 10.1038/nature10523 22031440 PMC3566780

[B42] Kathuria A. Lopez-Lengowski K. Watmuff B. Karmacharya R. (2020). Comparative transcriptomic analysis of cerebral organoids and cortical neuron cultures derived from human induced pluripotent stem cells. Stem Cells Dev. 29 (21), 1370–1381. 10.1089/scd.2020.0069 32862797 PMC7698857

[B43] Katsir K. W. Linial M. (2019). Human genes escaping X-inactivation revealed by single cell expression data. BMC Genomics 20 (1), 201. 10.1186/s12864-019-5507-6 30871455 PMC6419355

[B44] Khani F. Nafian S. Mollamohammadi S. Nemati S. Shokoohian B. Hassani S. N. (2022). Y chromosome genes may play roles in the development of neural rosettes from human embryonic stem cells. Stem Cell Rev. Rep. 18 (8), 3008–3020. 10.1007/s12015-022-10392-2 35661078

[B45] Kilpinen H. Goncalves A. Leha A. Afzal V. Alasoo K. Ashford S. (2017). Common genetic variation drives molecular heterogeneity in human iPSCs. Nature 546 (7658), 370–375. 10.1038/nature22403 28489815 PMC5524171

[B46] Kim D. Paggi J. M. Park C. Bennett C. Salzberg S. L. (2019). Graph-based genome alignment and genotyping with HISAT2 and HISAT-genotype. Nat. Biotechnol. 37 (8), 907–915. 10.1038/s41587-019-0201-4 31375807 PMC7605509

[B47] Kim J.-H. Lee J. H. Lee I. S. Lee S. B. Cho K. S. (2017). Histone lysine methylation and neurodevelopmental disorders. Int. J. Mol. Sci. 18 (7), 1404. 10.3390/ijms18071404 28665350 PMC5535897

[B48] Kim Y. Jeong Y. Kwon K. Ismail T. Lee H. K. Kim C. (2018). Physiological effects of KDM5C on neural crest migration and eye formation during vertebrate development. Epigenetics Chromatin 11 (1), 72. 10.1186/s13072-018-0241-x 30522514 PMC6282277

[B49] Kimura S. Lok J. Gelman I. H. Lo E. H. Arai K. (2023). Role of A-kinase anchoring protein 12 in the central nervous system. J. Clin. neurology (Seoul, Korea) 19 (4), 329–337. 10.3988/jcn.2023.0095 PMC10329933 37417430

[B50] Kuruş M. Akbari S. Eskier D. Bursalı A. Ergin K. Erdal E. (2021). Transcriptome dynamics of human neuronal differentiation from iPSC. Front. Cell Dev. Biol. 9, 727747. 10.3389/fcell.2021.727747 34970540 PMC8712770

[B51] Lan F. Bayliss P. E. Rinn J. L. Whetstine J. R. Wang J. K. Chen S. (2007). A histone H3 lysine 27 demethylase regulates animal posterior development. Nature 449 (7163), 689–694. 10.1038/nature06192 17851529

[B52] Lee M. G. Villa R. Trojer P. Norman J. Yan K. P. Reinberg D. (2007). Demethylation of H3K27 regulates polycomb recruitment and H2A ubiquitination. Science 318 (5849), 447–450. 10.1126/science.1149042 17761849

[B53] Lei X. Jiao J. (2018). UTX affects neural stem cell proliferation and differentiation through PTEN signaling. Stem Cell Rep. 10 (4), 1193–1207. 10.1016/j.stemcr.2018.02.008 PMC5998300 29551674

[B54] Liao Y. Smyth G. K. Shi W. (2014). featureCounts: an efficient general purpose program for assigning sequence reads to genomic features. Bioinformatics 30 (7), 923–930. 10.1093/bioinformatics/btt656 24227677

[B55] Lindhout F. W. Kooistra R. Portegies S. Herstel L. J. Stucchi R. Snoek B. L. (2020). Quantitative mapping of transcriptome and proteome dynamics during polarization of human iPSC-derived neurons. eLife 9, e58124. 10.7554/eLife.58124 32940601 PMC7498259

[B56] Liu S. Seidlitz J. Blumenthal J. D. Clasen L. S. Raznahan A. (2020). Integrative structural, functional, and transcriptomic analyses of sex-biased brain organization in humans. Proc. Natl. Acad. Sci. U. S. A. 117 (31), 18788–18798. 10.1073/pnas.1919091117 32690678 PMC7414084

[B57] Loke H. Harley V. Lee J. (2015). Biological factors underlying sex differences in neurological disorders. Int. J. Biochem. Cell Biol. 65, 139–150. 10.1016/j.biocel.2015.05.024 26028290

[B58] Love M. I. Huber W. Anders S. (2014). Moderated estimation of fold change and dispersion for RNA-seq data with DESeq2. Genome Biol. 15 (12), 550. 10.1186/s13059-014-0550-8 25516281 PMC4302049

[B59] Macarthur C. C. Xue H. Van Hoof D. Lieu P. T. Dudas M. Fontes A. (2012). Chromatin insulator elements block transgene silencing in engineered human embryonic stem cell lines at a defined chromosome 13 locus. Stem Cells Dev. 21 (2), 191–205. 10.1089/scd.2011.0163 21699412 PMC3258440

[B60] Maenner M. J. Shaw K. A. Baio J. Washington A. Patrick M. DiRienzo M. (2020). Prevalence of autism spectrum disorder among children aged 8 Years - autism and developmental disabilities monitoring network, 11 sites, United States, 2016. MMWR 69 (4), 1–12. 10.15585/mmwr.ss6904a1 PMC7119644 32214087

[B61] Main H. Hedenskog M. Acharya G. Hovatta O. Lanner F. (2020). Karolinska institutet human embryonic stem cell bank. Stem Cell Res. 45, 101810. 10.1016/j.scr.2020.101810 32339905

[B62] Mallard T. T. Liu S. Seidlitz J. Ma Z. Moraczewski D. Thomas A. (2021). X-chromosome influences on neuroanatomical variation in humans. Nat. Neurosci. 24 (9), 1216–1224. 10.1038/s41593-021-00890-w 34294918

[B63] Maroof A. M. Keros S. Tyson J. A. Ying S. W. Ganat Y. M. Merkle F. T. (2013). Directed differentiation and functional maturation of cortical interneurons from human embryonic stem cells. Cell Stem Cell 12 (5), 559–572. 10.1016/j.stem.2013.04.008 23642365 PMC3681523

[B64] May T. Adesina I. McGillivray J. Rinehart N. J. (2019). Sex differences in neurodevelopmental disorders. Curr. Opin. Neurology 32 (4), 622–626. 10.1097/WCO.0000000000000714 31135460

[B65] Mayer A. Lahr G. Swaab D. F. Pilgrim C. Reisert I. (1998). The Y-chromosomal genes SRY and ZFY are transcribed in adult human brain. Neurogenetics 1 (4), 281–288. 10.1007/s100480050042 10732804

[B66] McCarthy M. M. (2009). The two faces of estradiol: effects on the developing brain. Neurosci. 15 (6), 599–610. 10.1177/1073858409340924 PMC2795061 19700741

[B67] Meisig J. Dreser N. Kapitza M. Henry M. Rotshteyn T. Rahnenführer J. (2020). Kinetic modeling of stem cell transcriptome dynamics to identify regulatory modules of normal and disturbed neuroectodermal differentiation. Nucleic Acids Res. 48 (22), 12577–12592. 10.1093/nar/gkaa1089 33245762 PMC7736781

[B68] Meyfour A. Pooyan P. Pahlavan S. Rezaei-Tavirani M. Gourabi H. Baharvand H. (2017). Chromosome-centric human proteome project allies with developmental Biology: a case study of the role of Y chromosome genes in organ development. J. Proteome Res. 16 (12), 4259–4272. 10.1021/acs.jproteome.7b00446 28914051

[B69] Millan M. J. (2013). An epigenetic framework for neurodevelopmental disorders: from pathogenesis to potential therapy. Neuropharmacology 68, 2–82. 10.1016/j.neuropharm.2012.11.015 23246909

[B70] Mootha V. K. Lindgren C. M. Eriksson K. F. Subramanian A. Sihag S. Lehar J. (2003). PGC-1alpha-responsive genes involved in oxidative phosphorylation are coordinately downregulated in human diabetes. Nat. Genet. 34 (3), 267–273. 10.1038/ng1180 12808457

[B71] Motosugi N. Sugiyama A. Okada C. Otomo A. Umezawa A. Akutsu H. (2022). De-erosion of X chromosome dosage compensation by the editing of XIST regulatory regions restores the differentiation potential in hPSCs. Cell Rep. methods 2 (12), 100352. 10.1016/j.crmeth.2022.100352 36590687 PMC9795333

[B72] Najmabadi H. Hu H. Garshasbi M. Zemojtel T. Abedini S. S. Chen W. (2011). Deep sequencing reveals 50 novel genes for recessive cognitive disorders. Nature 478 (7367), 57–63. 10.1038/nature10423 21937992

[B73] Narsinh K. H. Plews J. Wu J. C. (2011). Comparison of human induced pluripotent and embryonic stem cells: fraternal or identical twins? Mol. Ther. 19 (4), 635–638. 10.1038/mt.2011.41 21455209 PMC3070108

[B74] Nguyen D. K. Disteche C. M. (2006). High expression of the mammalian X chromosome in brain. Brain Res. 1126 (1), 46–49. 10.1016/j.brainres.2006.08.053 16978591

[B75] Nguyen T. A. Wu K. Pandey S. Lehr A. W. Li Y. Bemben M. A. (2020). A cluster of autism-associated variants on X-linked NLGN4X functionally resemble NLGN4Y. Neuron 106 (5), 759–768. 10.1016/j.neuron.2020.03.008 32243781 PMC7491604

[B76] O’Brien H. E. Hannon E. Jeffries A. R. Davies W. Hill M. J. Anney R. J. (2018). Sex differences in gene expression in the human fetal brain. BioRxiv.

[B77] Oliva M. Muñoz-Aguirre M. Kim-Hellmuth S. Wucher V. Gewirtz A. D. H. Cotter D. J. (2020). The impact of sex on gene expression across human tissues. Science 369 (6509), eaba3066. 10.1126/science.aba3066 32913072 PMC8136152

[B78] Pancho A. Aerts T. Mitsogiannis M. D. Seuntjens E. (2020). Protocadherins at the crossroad of signaling pathways. Front. Mol. Neurosci. 13, 117. 10.3389/fnmol.2020.00117 32694982 PMC7339444

[B79] Pandya M. Palpagama T. H. Turner C. Waldvogel H. J. Faull R. L. Kwakowsky A. (2019). Sex- and age-related changes in GABA signaling components in the human cortex. Biol. sex Differ. 10 (1), 5. 10.1186/s13293-018-0214-6 30642393 PMC6332906

[B80] Panopoulos A. D. D'Antonio M. Benaglio P. Williams R. Hashem S. I. Schuldt B. M. (2017). iPSCORE: a resource of 222 iPSC lines enabling functional characterization of genetic variation across a variety of cell types. Stem Cell Rep. 8 (4), 1086–1100. 10.1016/j.stemcr.2017.03.012 PMC5390244 28410642

[B81] Poeta L. Fusco F. Drongitis D. Shoubridge C. Manganelli G. Filosa S. (2013). A regulatory path associated with X-linked intellectual disability and epilepsy links KDM5C to the polyalanine expansions in ARX. Am. J. Hum. Genet. 92 (1), 114–125. 10.1016/j.ajhg.2012.11.008 23246292 PMC3542471

[B82] Pottmeier P. Doszyn O. Peuckert C. Jazin E. (2020). Increased expression of Y-encoded demethylases during differentiation of human male neural stem cells. Stem Cells Dev. 29 (23), 1497–1509. 10.1089/scd.2020.0138 33040644

[B83] Prasad A. Merico D. Thiruvahindrapuram B. Wei J. Lionel A. C. Sato D. (2012). A discovery resource of rare copy number variations in individuals with autism spectrum disorder. G3 (Bethesda, Md.) 2 (12), 1665–1685. 10.1534/g3.112.004689 23275889 PMC3516488

[B84] Raznahan A. Disteche C. M. (2021). X-chromosome regulation and sex differences in brain anatomy. Neurosci. Biobehav. Rev. 120, 28–47. 10.1016/j.neubiorev.2020.10.024 33171144 PMC7855816

[B85] Raznahan A. Parikshak N. N. Chandran V. Blumenthal J. D. Clasen L. S. Alexander-Bloch A. F. (2018). Sex-chromosome dosage effects on gene expression in humans. Proc. Natl. Acad. Sci. U. S. A. 115 (28), 7398–7403. 10.1073/pnas.1802889115 29946024 PMC6048519

[B86] Ronan J. L. Wu W. Crabtree G. R. (2013). From neural development to cognition: unexpected roles for chromatin. Nat. Rev. Genet. 14 (5), 347–359. 10.1038/nrg3413 23568486 PMC4010428

[B87] Ronen D. Benvenisty N. (2014). Sex-dependent gene expression in human pluripotent stem cells. Cell Rep. 8 (4), 923–932. 10.1016/j.celrep.2014.07.013 25127145

[B88] Saha S. Chant D. Welham J. McGrath J. (2005). A systematic review of the prevalence of schizophrenia. PLoS Med. 2 (5), e141. 10.1371/journal.pmed.0020141 15916472 PMC1140952

[B89] Salomonis N. Dexheimer P. J. Omberg L. Schroll R. Bush S. Huo J. (2016). Integrated genomic analysis of diverse induced pluripotent stem cells from the progenitor cell Biology consortium. Stem Cell Rep. 7 (1), 110–125. 10.1016/j.stemcr.2016.05.006 PMC4944587 27293150

[B90] Sarel-Gallily R. Benvenisty N. (2022). Large-scale analysis of X inactivation variations between primed and naïve human embryonic stem cells. Cells 11 (11), 1729. 10.3390/cells11111729 35681423 PMC9179337

[B91] Schneider V. A. Graves-Lindsay T. Howe K. Bouk N. Chen H. C. Kitts P. A. (2017). Evaluation of GRCh38 and *de novo* haploid genome assemblies demonstrates the enduring quality of the reference assembly. Genome Res. 27 (5), 849–864. 10.1101/gr.213611.116 28396521 PMC5411779

[B92] Schwartzentruber J. Foskolou S. Kilpinen H. Rodrigues J. Alasoo K. Knights A. J. (2018). Molecular and functional variation in iPSC-derived sensory neurons. Nat. Genet. 50 (1), 54–61. 10.1038/s41588-017-0005-8 29229984 PMC5742539

[B93] Shan Y. Zhang Y. Zhao Y. Wang T. Zhang J. Yao J. (2020). JMJD3 and UTX determine fidelity and lineage specification of human neural progenitor cells. Nat. Commun. 11 (1), 382. 10.1038/s41467-019-14028-x 31959746 PMC6971254

[B94] Shannon P. Markiel A. Ozier O. Baliga N. S. Wang J. T. Ramage D. (2003). Cytoscape: a software environment for integrated models of biomolecular interaction networks. Genome Res. 13 (11), 2498–2504. 10.1101/gr.1239303 14597658 PMC403769

[B95] Sherman B. T. Hao M. Qiu J. Jiao X. Baseler M. W. Lane H. C. (2022). DAVID: a web server for functional enrichment analysis and functional annotation of gene lists (2021 update). Nucleic Acids Res. 50 (1), W216–W221. 10.1093/nar/gkac194 35325185 PMC9252805

[B96] Shi L. Zhang Z. Su B. (2016). Sex biased gene expression profiling of human brains at major developmental stages. Sci. Rep. 6, 21181. 10.1038/srep21181 26880485 PMC4754746

[B97] Shpargel K. B. Sengoku T. Yokoyama S. Magnuson T. (2012). UTX and UTY demonstrate histone demethylase-independent function in mouse embryonic development. PLoS Genet. 8 (9), e1002964. 10.1371/journal.pgen.1002964 23028370 PMC3459986

[B98] Shulha H. P. Cheung I. Guo Y. Akbarian S. Weng Z. (2013). Coordinated cell type-specific epigenetic remodeling in prefrontal cortex begins before birth and continues into early adulthood. PLoS Genet. 9 (4), e1003433. 10.1371/journal.pgen.1003433 23593028 PMC3623761

[B99] Sidorenko J. Kassam I. Kemper K. E. Zeng J. Lloyd-Jones L. R. Montgomery G. W. (2019). The effect of X-linked dosage compensation on complex trait variation. Nat. Commun. 10 (1), 3009. 10.1038/s41467-019-10598-y 31285442 PMC6614401

[B100] Sismani C. Anastasiadou V. Kousoulidou L. Parkel S. Koumbaris G. Zilina O. (2011). 9 Mb familial duplication in chromosome band Xp22.2-22.13 associated with mental retardation, hypotonia and developmental delay, scoliosis, cardiovascular problems and mild dysmorphic facial features. Eur. J. Med. Genet. 54 (5), e510–e515. 10.1016/j.ejmg.2011.05.006 21684358

[B101] Skuse D. H. (2005). X-linked genes and mental functioning. Hum. Mol. Genet. 14 (1), R27–R32. 10.1093/hmg/ddi112 15809269

[B102] Solomon E. Davis-Anderson K. Hovde B. Micheva-Viteva S. Harris J. F. Twary S. (2021). Global transcriptome profile of the developmental principles of *in vitro* iPSC-to-motor neuron differentiation. BMC Mol. Cell Biol. 22 (1), 13. 10.1186/s12860-021-00343-z 33602141 PMC7893891

[B103] Song Y. Botvinnik O. B. Lovci M. T. Kakaradov B. Liu P. Xu J. L. (2017). Single-cell alternative splicing analysis with expedition reveals splicing dynamics during neuron differentiation. Mol. Cell 67 (1), 148–161. 10.1016/j.molcel.2017.06.003 28673540 PMC5540791

[B104] Strano A. Tuck E. Stubbs V. E. Livesey F. J. (2020). Variable outcomes in neural differentiation of human PSCs arise from intrinsic differences in developmental signaling pathways. Cell Rep. 31 (10), 107732. 10.1016/j.celrep.2020.107732 32521257 PMC7296348

[B105] Strømme P. Mangelsdorf M. E. Scheffer I. E. Gécz J. (2002). Infantile spasms, dystonia, and other X-linked phenotypes caused by mutations in Aristaless related homeobox gene, ARX. Brain & Dev. 24 (5), 266–268. 10.1016/s0387-7604(02)00079-7 12142061

[B106] Subramanian A. Tamayo P. Mootha V. K. Mukherjee S. Ebert B. L. Gillette M. A. (2005). Gene set enrichment analysis: a knowledge-based approach for interpreting genome-wide expression profiles. Proc. Natl. Acad. Sci. U. S. A. 102 (43), 15545–15550. 10.1073/pnas.0506580102 16199517 PMC1239896

[B107] Swigut T. Wysocka J. (2007). H3K27 demethylases, at long last. Cell 131 (1), 29–32. 10.1016/j.cell.2007.09.026 17923085

[B108] Tang Q.-Y. Zhang S. F. Dai S. K. Liu C. Wang Y. Y. Du H. Z. (2020). UTX regulates human neural differentiation and dendritic morphology by resolving bivalent promoters. Stem Cell Rep. 15 (2), 439–453. 10.1016/j.stemcr.2020.06.015 PMC7419705 32679064

[B109] Tesic A. Rodgers S. Müller M. Wagner E. Y. N. von Känel R. Castelao E. (2019). Sex differences in neurodevelopmental and common mental disorders examined from three epidemiological perspectives. Psychiatry Res. 278, 213–217. 10.1016/j.psychres.2019.06.019 31226547

[B110] Thomson J. A. Itskovitz-Eldor J. Shapiro S. S. Waknitz M. A. Swiergiel J. J. Marshall V. S. (1998). Embryonic stem cell lines derived from human blastocysts. Science 282 (5391), 1145–1147. 10.1126/science.282.5391.1145 9804556

[B111] Trabzuni D. Ramasamy A. Imran S. Walker R. Smith C. Weale M. E. (2013). Widespread sex differences in gene expression and splicing in the adult human brain. Nat. Commun. 4, 2771. 10.1038/ncomms3771 24264146 PMC3868224

[B112] Tsankov A. M. Akopian V. Pop R. Chetty S. Gifford C. A. Daheron L. (2015). A qPCR ScoreCard quantifies the differentiation potential of human pluripotent stem cells. Nat. Biotechnol. 33 (11), 1182–1192. 10.1038/nbt.3387 26501952 PMC4636964

[B113] Tukiainen T. Villani A. C. Yen A. Rivas M. A. Marshall J. L. Satija R. (2017). Landscape of X chromosome inactivation across human tissues. Nature 550 (7675), 244–248. 10.1038/nature24265 29022598 PMC5685192

[B114] Vakilian H. Mirzaei M. Sharifi Tabar M. Pooyan P. Habibi Rezaee L. Parker L. (2015). DDX3Y, a male-specific region of Y chromosome gene, may modulate neuronal differentiation. J. Proteome Res. 14 (9), 3474–3483. 10.1021/acs.jproteome.5b00512 26144214

[B115] van de Leemput J. Boles N. C. Kiehl T. R. Corneo B. Lederman P. Menon V. (2014). CORTECON: a temporal transcriptome analysis of *in vitro* human cerebral cortex development from human embryonic stem cells. Neuron 83 (1), 51–68. 10.1016/j.neuron.2014.05.013 24991954

[B116] Verma A. Kommaddi R. P. Gnanabharathi B. Hirsch E. C. Ravindranath V. (2023). Genes critical for development and differentiation of dopaminergic neurons are downregulated in Parkinson’s disease. J. Neural Transm. 130 (4), 495–512. 10.1007/s00702-023-02604-x 36820885

[B117] Villaescusa J. C. Li B. Toledo E. M. Rivetti di Val Cervo P. Yang S. Stott S. R. (2016). A PBX1 transcriptional network controls dopaminergic neuron development and is impaired in Parkinson’s disease. EMBO J. 35 (18), 1963–1978. 10.15252/embj.201593725 27354364 PMC5282836

[B118] Waldhorn I. Turetsky T. Steiner D. Gil Y. Benyamini H. Gropp M. (2022). Modeling sex differences in humans using isogenic induced pluripotent stem cells. Stem Cell Rep. 17 (12), 2732–2744. 10.1016/j.stemcr.2022.10.017 PMC9768579 36427492

[B119] Walport L. J. Hopkinson R. J. Vollmar M. Madden S. K. Gileadi C. Oppermann U. (2014). Human UTY(KDM6C) is a male-specific Nϵ-methyl lysyl demethylase. J. Biol. Chem. 289 (26), 18302–18313. 10.1074/jbc.M114.555052 24798337 PMC4140284

[B120] Warling A. Yavi M. Clasen L. S. Blumenthal J. D. Lalonde F. M. Raznahan A. (2021). Sex chromosome dosage effects on white matter structure in the human brain. Cereb. Cortex 31 (12), 5339–5353. 10.1093/cercor/bhab162 34117759 PMC8568008

[B121] Weickert C. S. Elashoff M. Richards A. B. Sinclair D. Bahn S. Paabo S. (2009). Transcriptome analysis of male-female differences in prefrontal cortical development. Mol. Psychiatry 14 (6), 558–561. 10.1038/mp.2009.5 19455171

[B122] Wigerius M. Quinn D. Fawcett J. P. (2020). Emerging roles for angiomotin in the nervous system. Sci. Signal. 13 (655), eabc0635. 10.1126/scisignal.abc0635 33109746

[B123] Wijchers P. J. Yandim C. Panousopoulou E. Ahmad M. Harker N. Saveliev A. (2010). Sexual dimorphism in mammalian autosomal gene regulation is determined not only by Sry but by sex chromosome complement as well. Dev. Cell 19 (3), 477–484. 10.1016/j.devcel.2010.08.005 20833369

[B124] Wu J. Q. Habegger L. Noisa P. Szekely A. Qiu C. Hutchison S. (2010). Dynamic transcriptomes during neural differentiation of human embryonic stem cells revealed by short, long, and paired-end sequencing. Proc. Natl. Acad. Sci. U. S. A. 107 (11), 5254–5259. 10.1073/pnas.0914114107 20194744 PMC2841935

[B125] Yang X. Xu B. Mulvey B. Evans M. Jordan S. Wang Y. D. (2019). Differentiation of human pluripotent stem cells into neurons or cortical organoids requires transcriptional co-regulation by UTX and 53BP1. Nat. Neurosci. 22 (3), 362–373. 10.1038/s41593-018-0328-5 30718900 PMC6511450

[B126] Zagni E. Simoni L. Colombo D. (2016). Sex and gender differences in central nervous system-related disorders. Neurosci. J. 2016, 2827090. 10.1155/2016/2827090 27314003 PMC4904110

[B127] Zhang Y. Castillo-Morales A. Jiang M. Zhu Y. Hu L. Urrutia A. O. (2013). Genes that escape X-inactivation in humans have high intraspecific variability in expression, are associated with mental impairment but are not slow evolving. Mol. Biol. Evol. 30 (12), 2588–2601. 10.1093/molbev/mst148 24023392 PMC3840307

