# Supplementary material for: Sex-biased gene expression during neural differentiation of human embryonic stem cells
Source: Front Cell Dev Biol. 2024 May 1;12:1341373. doi: 10.3389/fcell.2024.1341373 (PMC11101176; doi:10.3389/fcell.2024.1341373)
Supplement: Supplementary file 2 [file DataSheet1.docx]

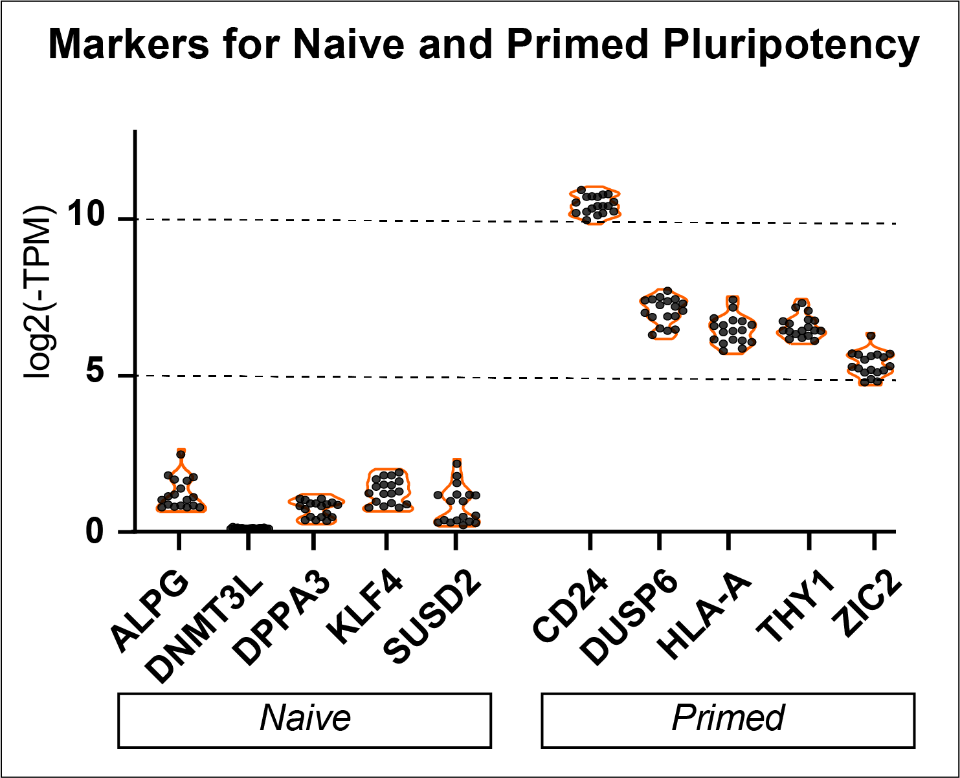


Supplementary Figure 1**:** Gene expression markers for naive and primer pluripotency at D0, before differentiation. Each dot represents one of three experimental replicates of all 6 cell lines investigated by RNA sequencing.


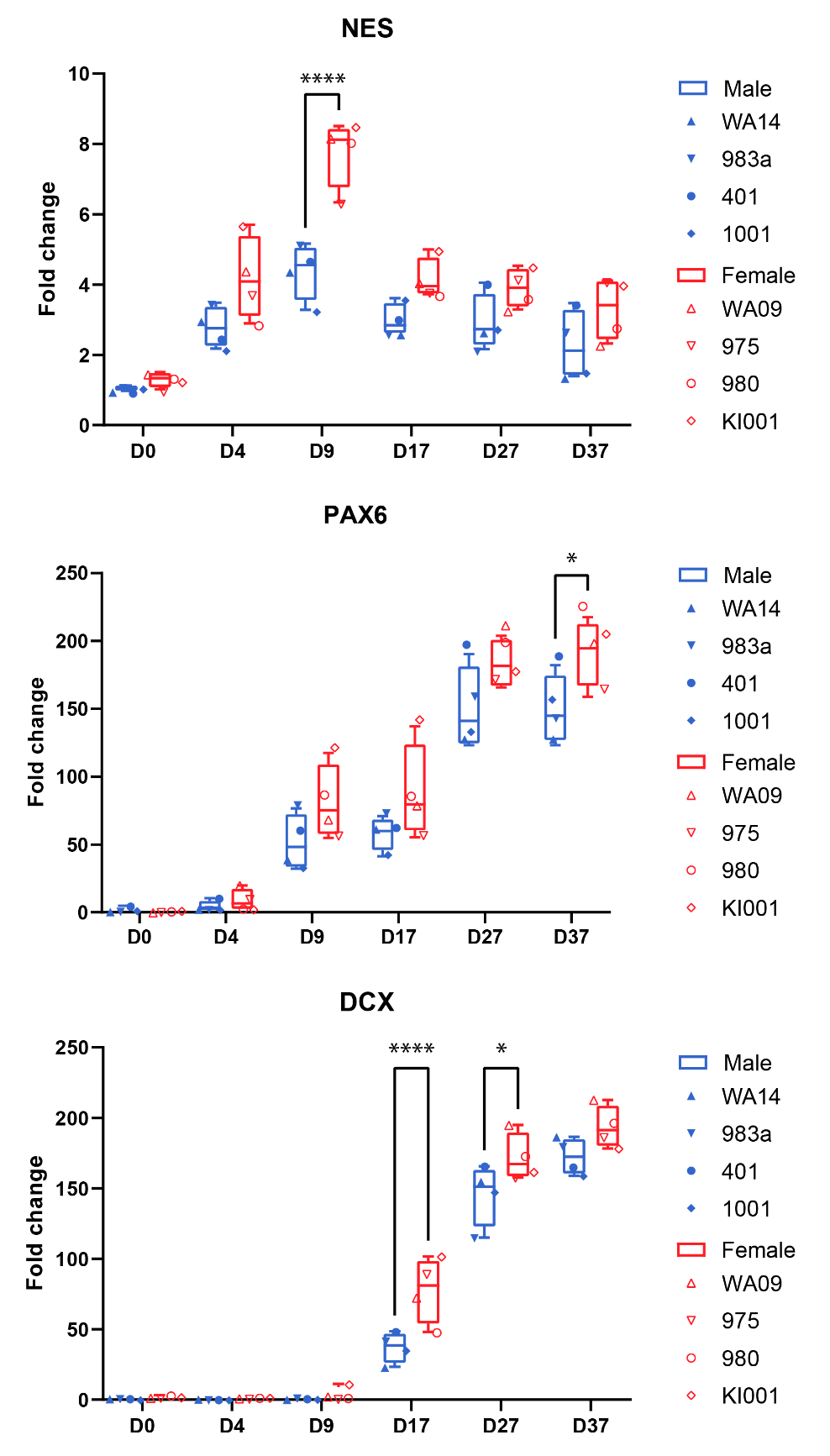


**Supplementary Figure 2:** qPCR analysis of NES, PAX6 and DCX in repetitions of the 37 day neural differentiation experiment (n=3). Sex-biased expression was noticed in NES at D9 (p<0.0001), PAX6 at D37 (p<0.05) and DCX at D17 (p<0.001) using a repeated measurements two-way ANOVA with Šidák’s multiple comparisons test.


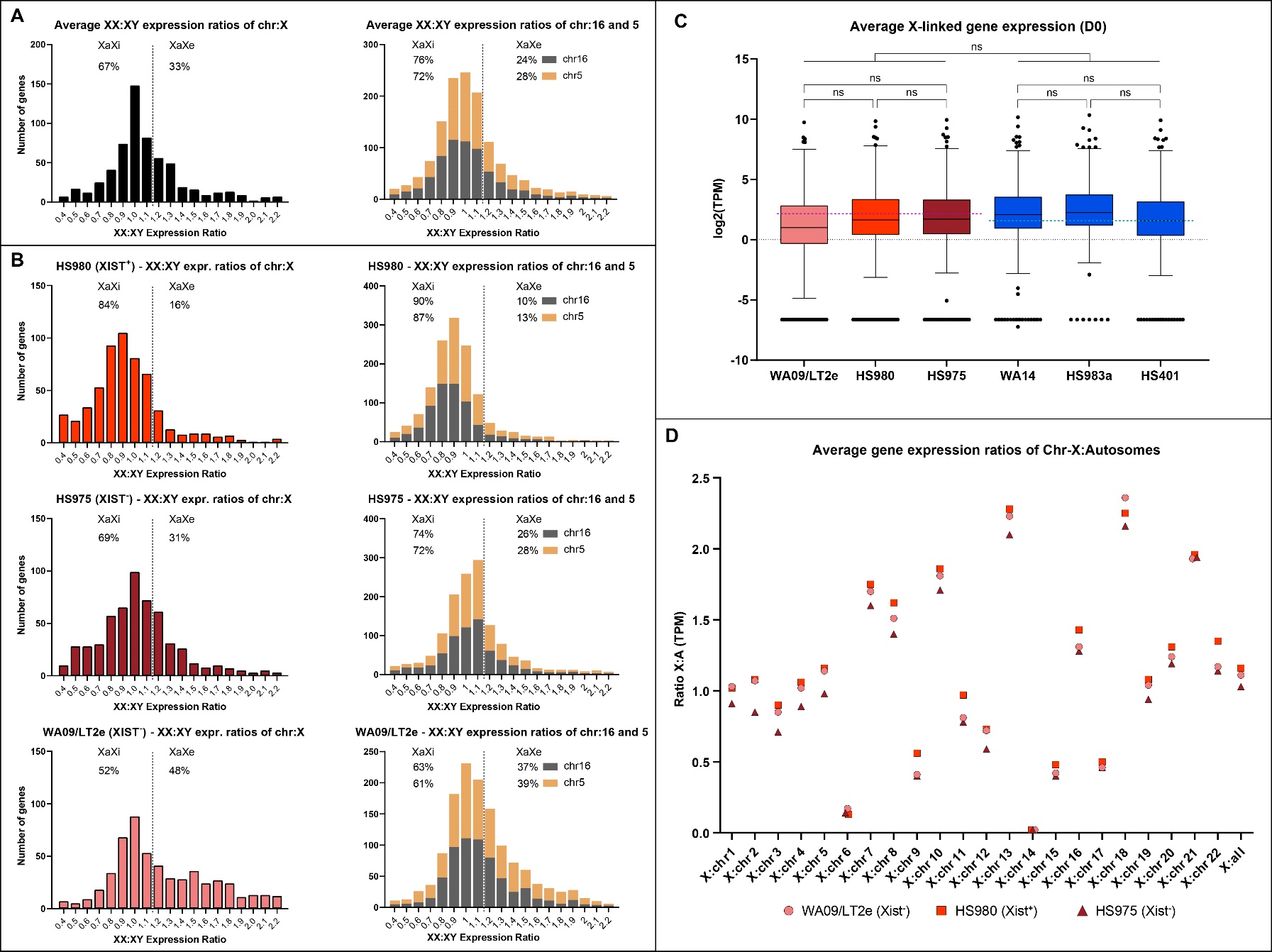
**Supplementary Figure 3: (A)** Average XX:XY gene expression ratio of X-linked genes. Genes with a ratio of >1.1 are considered to be affected by XCI erosion. **(B)** XX:XY gene expression ratios of chromosome 5 and 16 were used as a control to estimate the baseline expression ratio in XX and XY cell lines. **(C)** Average gene expression of chromosome X of male and female cell lines at D0. No significant difference was detected between the averages using a one-way ANOVA with Tukey’s multiple comparison test. **(D)** Average gene expression ratios of X chromosomal and autosomal genes displaying subtle inclination towards an increased autosomal gene expression in the Xist-positive cell line HS980. Notably on chromosomes 8, 9, 11 and 22.


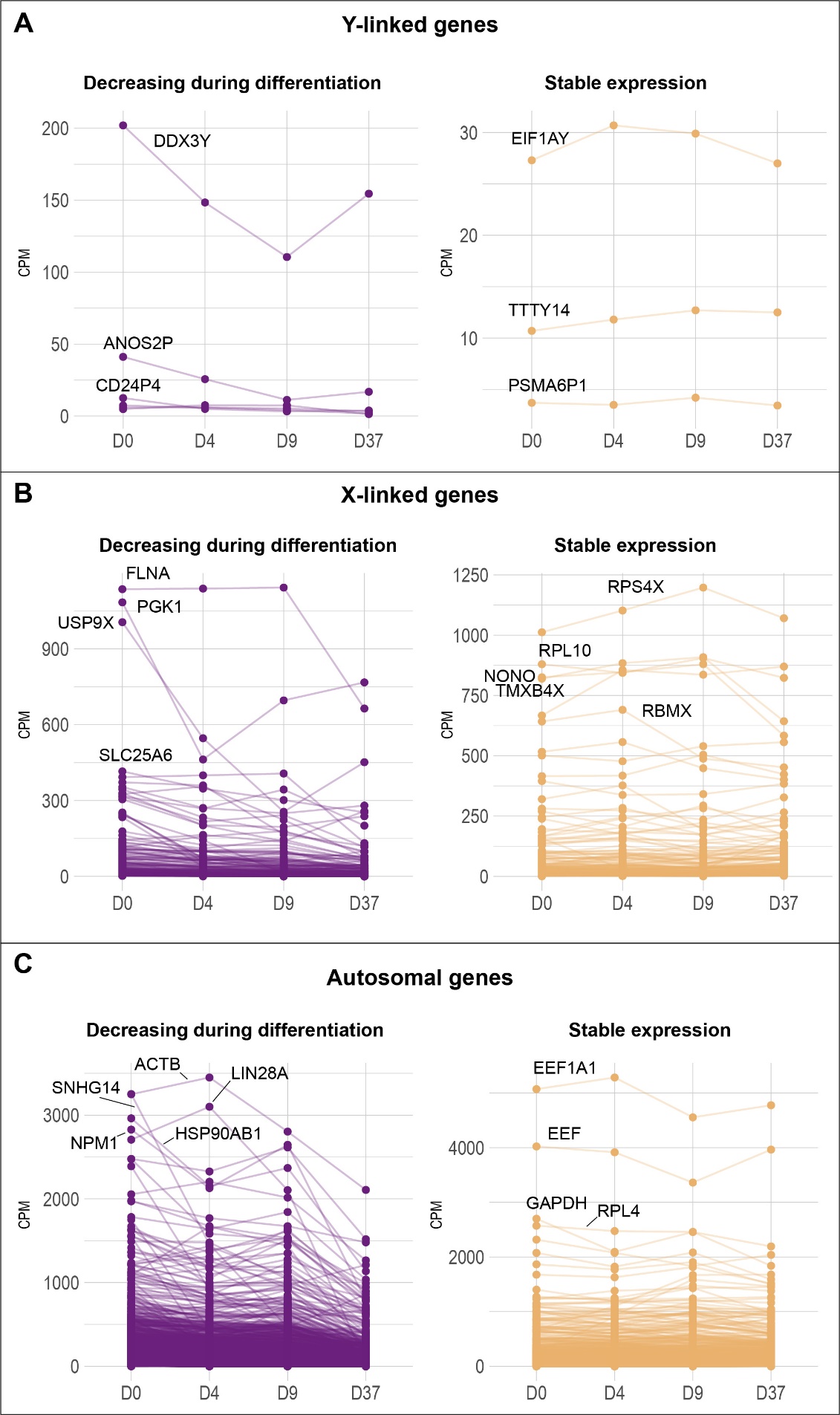


**Supplementary Figure 4:** Decreasing and stable gene expression of **(A)** Y‑linked, **(B)** X-linked and **(C)** autosomal genes during neural differentiation (D0, D4, D9, D37).


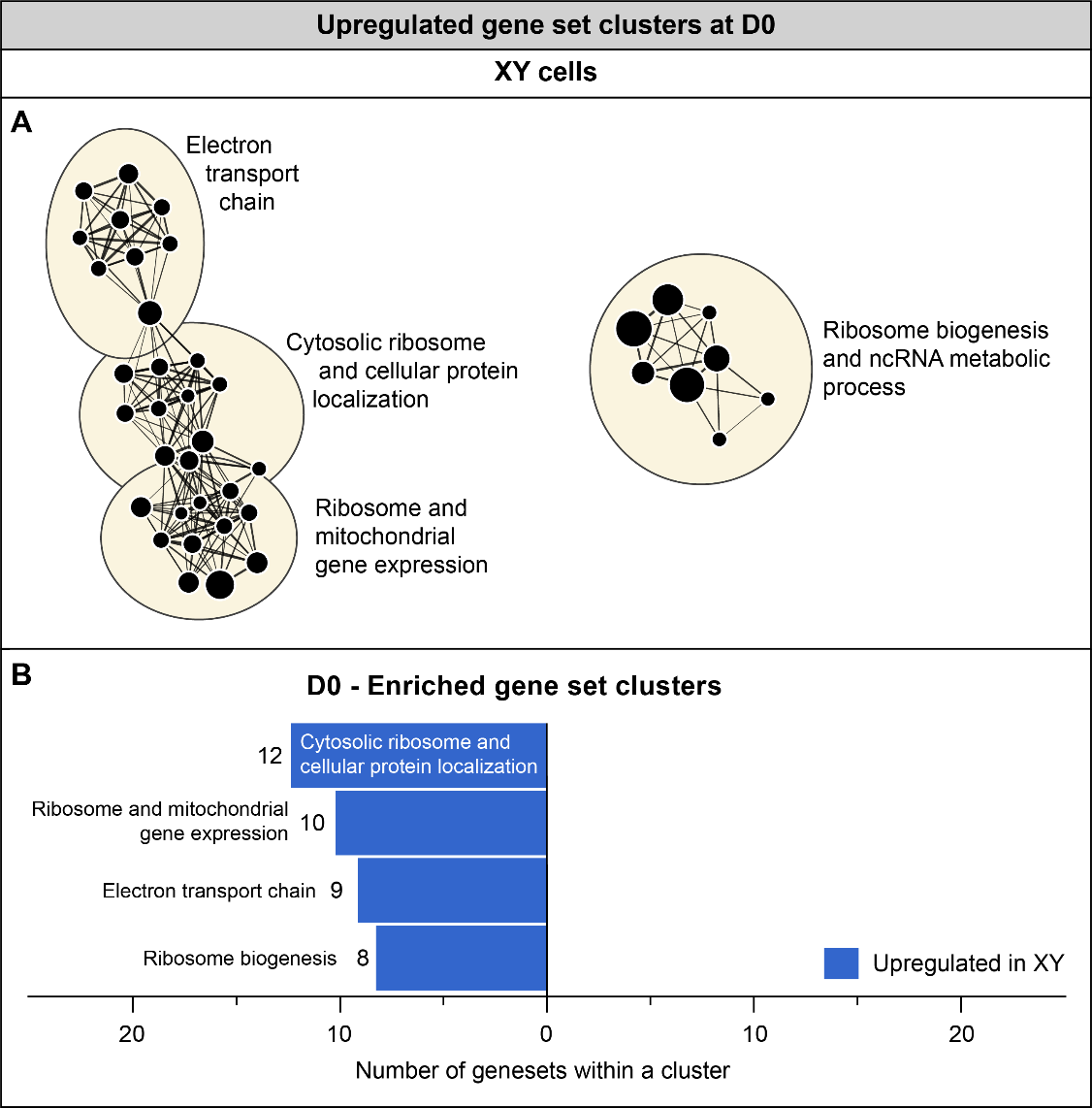


**Supplementary Figure 5:** Gene set enrichment analysis based on differentially expressed genes at D0, before the start of differentiation. **A)** Enrichment map, clusters of gene sets of the same function are connected and in close proximity. **B)** Number of gene sets within a gene set cluster. Gene sets are filtered by an FDR q-value of <0.05.


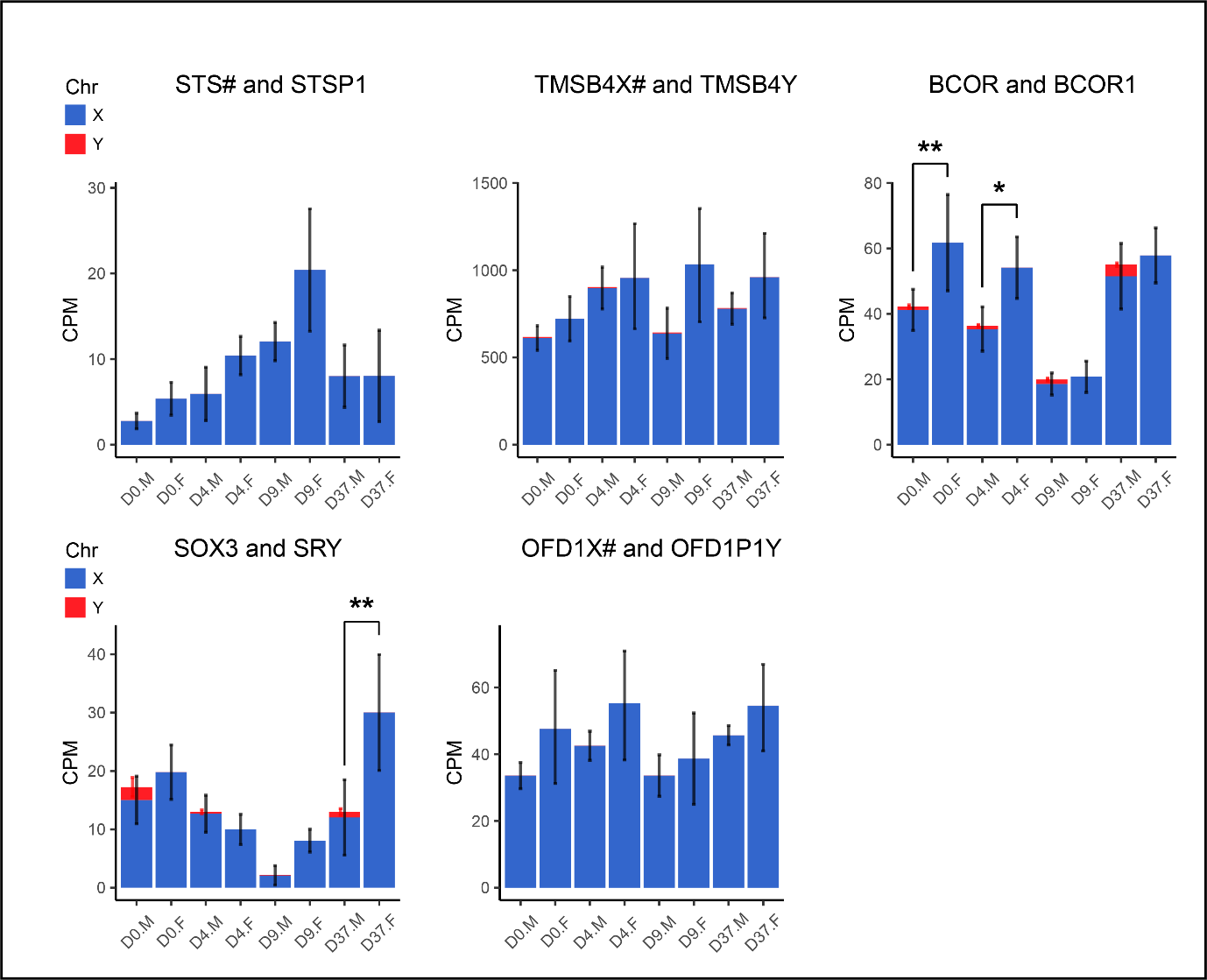


**Supplementary Figure 6:** X/Y homolog expression based on total RNA sequencing data (3 male and 3 female cell lines). A hashtag symbol after the X homolog indicates that the gene is escaping XCI. The homologous gene pairs STS/P1, TMSB4X/Y, BCOR/1, SOX3/SRY and OFD1X/P1Y do not show significant changes during the neural differentiation period D0 to D37. Sex differences in gene dosage was found, with an increased gene dosage in female cell lines, in the gene pair BCOR/1 at D0 (p<0.01) and D4 (p<0.05) as well as in SOX3/SRY at D37 (p<0.01). Differences between the male and female samples were measured using repeated measurements two-way ANOVA with Šidák’s multiple comparisons test.


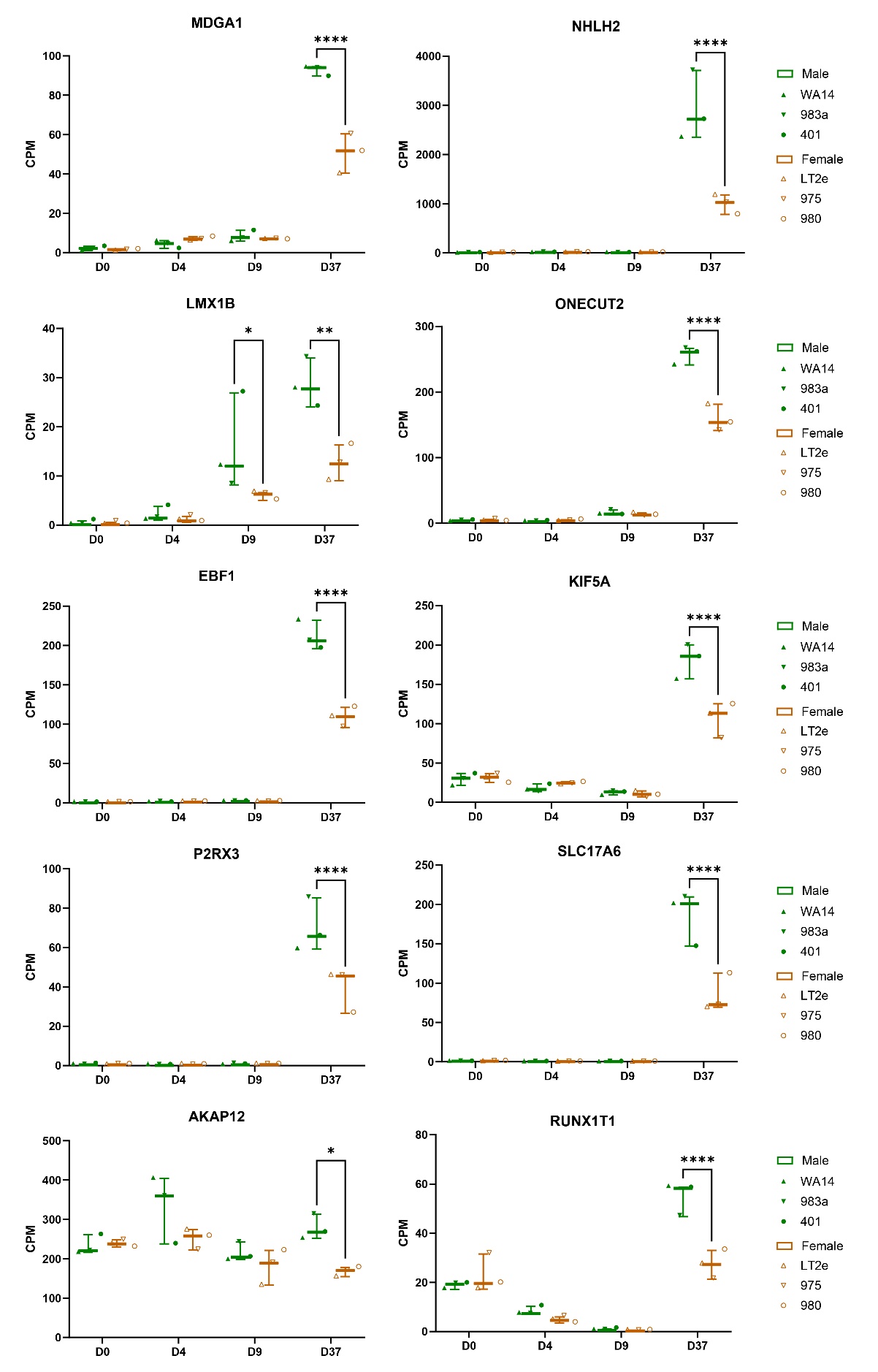


**Supplementary Figure 7:** Gene expression data of candidate genes from the three male (WA14, HS984a, HS401) and three female cell lines (WA09/LT2e, HS975, HS980) analyzed by RNA sequencing. The increased in gene expression is consistent with the findings obtained by qPCR (**Figure 6**). Statistically significant differences between male and female samples are demonstrated by repeated measurements ANOVA and Šidák’s multiple comparison test.


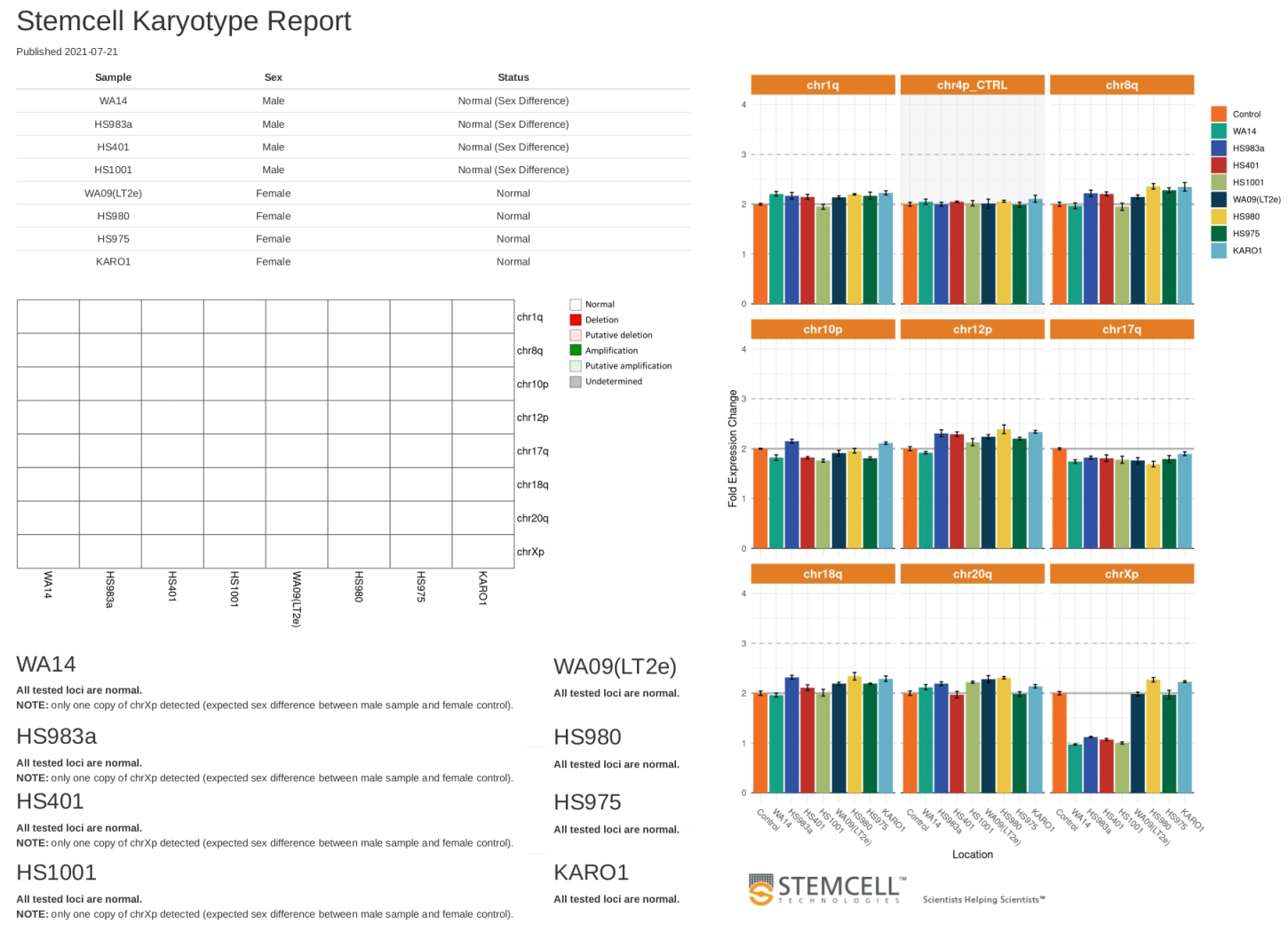


**Supplementary Figure 8:** Copy number variation analysis via qPCR to detect karyotypic abnormalities. The assay 'hPSC Genetic Analysis Kit' from STEMCELL Technologies (#07550) detects the 8 most common mutated regions in cell cultures. Including regions on chromosome 1, 8, 10, 12, 17, 18, 20 and X. No abnormalities were detected in the cell lines used in this study.
